# Single-nucleus multi-omics implicates androgen receptor signaling in cardiomyocytes and NR4A1 regulation in fibroblasts during atrial fibrillation

**DOI:** 10.1038/s44161-025-00626-0

**Published:** 2025-03-25

**Authors:** Francis J. A. Leblanc, Chi Him Kendrick Yiu, Lucia M. Moreira, Aaron M. Johnston, Neelam Mehta, Antonios Kourliouros, Rana Sayeed, Stanley Nattel, Svetlana Reilly, Guillaume Lettre

**Affiliations:** 1 Montreal Heart Institute, Montreal, Quebec Canada; 2 Department of Medicine, Université de Montréal, Montréal, Quebec Canada; 3 Division of Cardiovascular Medicine, Radcliffe Department of Medicine, University of Oxford, John Radcliffe Hospital, Oxford, UK; 4 Cardiothoracic Surgery, Oxford Heart Centre, John Radcliffe Hospital, Oxford, UK; 5 IHU Liryc and Fondation Bordeaux Université, Bordeaux, France; 6 Institute of Pharmacology, West German Heart and Vascular Center, Faculty of Medicine, University Duisburg-Essen, Essen, Germany

**Keywords:** Cell signalling, Transcriptomics

## Abstract

The dysregulation of gene expression programs in the human atria during persistent atrial fibrillation (AF) is not completely understood. Here, we reanalyze bulk RNA-sequencing datasets from two studies (*N* = 242) and identified 755 differentially expressed genes in left atrial appendages of individuals with persistent AF and non-AF controls. We combined the bulk RNA-sequencing differentially expressed genes with a left atrial appendage single-nucleus multi-omics dataset to assign genes to specific atrial cell types. We found noncoding genes at the *IFNG* locus (*LINC01479*, *IFNG-AS1*) strongly dysregulated in cardiomyocytes. We defined a gene expression signature potentially driven by androgen receptor signaling in cardiomyocytes from individuals with AF. Cell-type-specific gene expression modules suggested an increase in T cell and a decrease in adipocyte and neuronal cell gene expression in AF. Lastly, we showed that reducing NR4A1 expression, a marker of a poorly characterized human atrial fibroblast subtype, fibroblast activation markers, extracellular matrix remodeling and cell proliferation decreased.

## Abstract (web summary)

By analyzing bulk and single-cell RNA-sequencing datasets generated from left atrial appendages of individuals with atrial fibrillation and non-affected controls, Leblanc and Yiu identify cell-type-specific genes and transcriptomic programs implicated in atrial fibrillation, a cardiomyocyte-specific androgen receptor signaling signature and an anti-fibrotic effect of NR4A1 inhibition in atrial cardiofibroblasts.

## Main

AF is the most common arrhythmia, with an estimated global prevalence of 60 million cases^1,2^. Its prevalence is expected to double in the United States by 2050, and will reach 18 million in Europe by 2060 (refs. ^1,2^). This increase is mostly due to population aging, as age is the strongest risk factor for AF. To date, genetic studies of AF have almost exclusively implicated cardiomyocytes (CMs) as the causal driver cell type of the disease. Rare mutations in individuals with familial AF are largely found in ion channels, cardiac transcription factors (TFs) or cytoskeleton-associated proteins^3^. Genome-wide association studies of AF are specifically enriched for CM noncoding regulatory sequences^4^.

On the mechanistic level, numerous studies have implicated processes like fibrosis and inflammation in AF development and progression^3^. Atrial fibrosis, characterized by the accumulation of extracellular matrix (ECM) proteins, including type I collagen and type III collagen, is a hallmark of structural remodeling in AF. Atrial fibrosis, commonly coexisting with electrical remodeling, is a crucial arrhythmogenic substrate, driving AF perpetuation and the phenotype transition from paroxysmal to persistent AF, which notably hampers AF treatment. Currently, there are no available clinically effective drug targets for atrial fibrosis, highlighting the need to uncover new signaling molecules involved in AF pathogenesis. Furthermore, existing treatment of AF, including pharmacological therapies and catheter ablation, is suboptimal and associated with adverse side effects^3^. To identify novel therapeutic targets, many groups have conducted differential gene expression studies of the atria from AF and sinus rhythm (SR, controls) individuals. Most of these studies had small sample size, were limited to coding genes using microarray probes or were focused on differences across left and right atrial chambers^5^. Consequently, few genes are robustly differentially expressed in human AF^6^. Furthermore, the cell types and TFs responsible for all transcriptomic changes that occur during AF remodeling in human atrial tissue remain elusive.

Here, we sought to characterize robust differentially expressed genes (DEGs) in persistent AF at the cellular level and identify TFs responsible for this dysregulation. To maximize discovery power to refine DEGs while increasing cellular resolution, we combined bulk RNA-sequencing (RNA-seq) datasets and results from the 10x multiome assay (paired single-nucleus RNA sequencing (snRNA-seq) and open chromatin (single-nucleus assay for transposase-accessible chromatin sequencing (snATAC-seq)) in the same nuclei) to profile left atrial appendages (LAAs) from individuals with persistent AF and controls in SR. We identified cell-type-specific DEGs and modules, as well as a robust CM-specific AF DEG signature that implicates androgen receptor signaling. Finally, we identified *NR4A1* as one of the most specific gene markers of a poorly characterized subtype of atrial fibroblasts (FBs) and assessed its functions in human atrial FBs.

## Results

### Cell-type identification in human LAAs

**Fig. 1:**
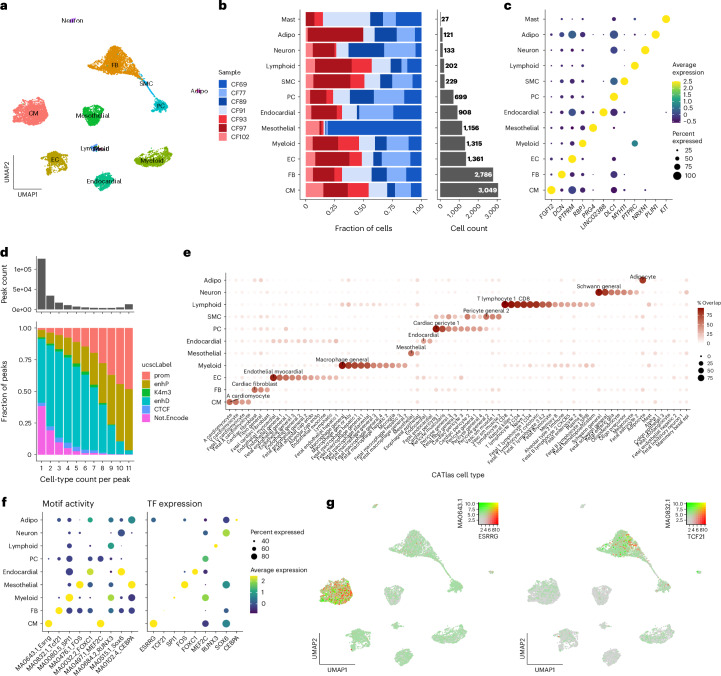
Single-nucleus multi-omics profiling to identify cell types in LAAs. **a**, Each cell type is colored differently in the uniform manifold approximation and projection (UMAP). **b**, The bar plots show the fraction of sample (left) and nucleus count (right) by cell type. Red, AF; Blu, SR. **c**, Cell-type expression of key marker genes. **d**, The histogram at the top represents the distribution of ATAC-seq peak count given how many cell types each peak is measured in. The bar plot at the bottom represents the fraction of ATAC-seq peaks located in ENCODE cCREs given how many cell types each peak is measured in. **e**, Comparison of the ATAC-seq peaks between cell types found in the scAF dataset and the Human Enhancer Atlas. We labeled cell types with the best ATAC-seq peak overlaps. **f**, Activity of the TF motif (from snATAC-seq, left) and expression of the corresponding TF (from snRNA-seq, right) in each cell type. **g**, UMAP of motif activity (green) and expression level (red) for two TFs: ESRRG on the left and TCF21 on the right. Adipo, adipocytes; CTCF, CCCTC-binding factor mark; enhD, distal enhancer; enhP, proximal enhancer; K4m3, lysine 4 tri-methyl mark; not.Encode, peak found in the scAF dataset without any overlapping ENCODE cCREs; PC, pericyte; Prom, promoter; SMC, smooth muscle cell.

To better understand the dysregulation of gene expression regulatory networks that occurs in persistent AF as well as the TFs responsible for these changes, we profiled RNA and open chromatin sites using 10x multiome snRNA-seq and ATAC-seq in nuclei of LAAs from four individuals with persistent AF and four controls in SR. The clinical characteristics of individuals are shown in Supplementary Table 1. After stringent quality control, we obtained a dataset composed of 11,986 nuclei (Supplementary Figs. 1–5) from seven samples (three AF and four SR). Hereafter, we refer to this dataset as ‘scAF’. We annotated cell types by co-embedding the scAF LAA nuclei with the human Heart Atlas left atrial nuclei^7^, identifying 12 major cell types (Supplementary Fig. 3c,d). Using dimensionality reduction techniques (principal component analysis (PCA) and latent semantic indexing for RNA-seq and ATAC-seq, respectively), we showed that clustering the scAF dataset without the Heart Atlas data recapitulates the same cell-type-specific clusters (Fig. 1a and Supplementary Fig. 4c,d). In agreement with other single-nucleus/single-cell human cardiac datasets^7,8^, CMs and FBs are the two most abundant cell types in scAF, accounting for 25% and 23% of all nuclei, respectively (Fig. 1b). We further validated cell-type identities using the expression pattern of known gene markers (CMs: *FGF12*, *TTN*, *RYR2*; FBs: *DCN*, *PTPRM*; endothelial cells (ECs): *PECAM1*; Fig. 1c, Supplementary Fig. 3 and Supplementary Table 2).

### The open chromatin landscape of human LAAs

Open chromatin sites identified in the scAF dataset were validated by comparing the called ATAC-seq peaks (*n* = 212,084, referred to as ‘peaks’) to the cross-tissue/cell-type ENCODE candidate *cis*-regulatory elements (cCREs)^9^. The majority (154,801, 73%) of peaks overlapped with ENCODE cCREs and were specific for one cell type (124,843, 59%). The number of cell types in which a peak was called was strongly associated with the ENCODE cCRE types (Fig. 1d). As reported previously^10^, promoters were typically accessible in multiple cell types, while distal enhancers exhibited greater cell-type specificity. Moreover, peaks that did not map to ENCODE cCREs, possibly because they are unique to adult atrial tissue, were more likely to be cell-type specific and displayed features characteristic of distal regulatory elements, for example, higher cell-type specificity, and reduced read counts, length and GC content (Supplementary Fig. 6). The peaks were then compared against the Human Enhancer Atlas, comprising 222 cell types derived from 30 adult and 15 fetal tissues, to exclude the possibility that the peaks were false positives^4^. This analysis that revealed the largest overlaps between cell-type-specific peaks in the scAF dataset and enhancers within the orthogonal dataset were always assigned to analogous cell types (Fig. 1e). For instance, FB-specific peaks had the highest overlap with adult cardiac FBs, the CM-specific peaks with adult atrial CMs and the EC-specific peaks with cardiac ECs (Fig. 1e). Finally, we examined TF motif activities by cell type using ChromVar^11^. Motifs belonging to the same family often produced similar scores, which is unsurprising given the similarity of their motifs. To circumvent this limitation, we leveraged the paired modalities of our scAF dataset to select TFs that are expressed (snRNA-seq) and have active binding motifs (snATAC-seq) in the same cell type (Fig. 1f and Supplementary Table 3). With this approach, we prioritized TFs with known roles in the heart, such as *ESRRG* and *TBX5* in CM^12,13^, and *TCF21* in FBs^14^ (Fig. 1g and Supplementary Table 3).

### Combining RNA-seq datasets to assign DEGs to cell types

**Extended Data Fig. 1:**
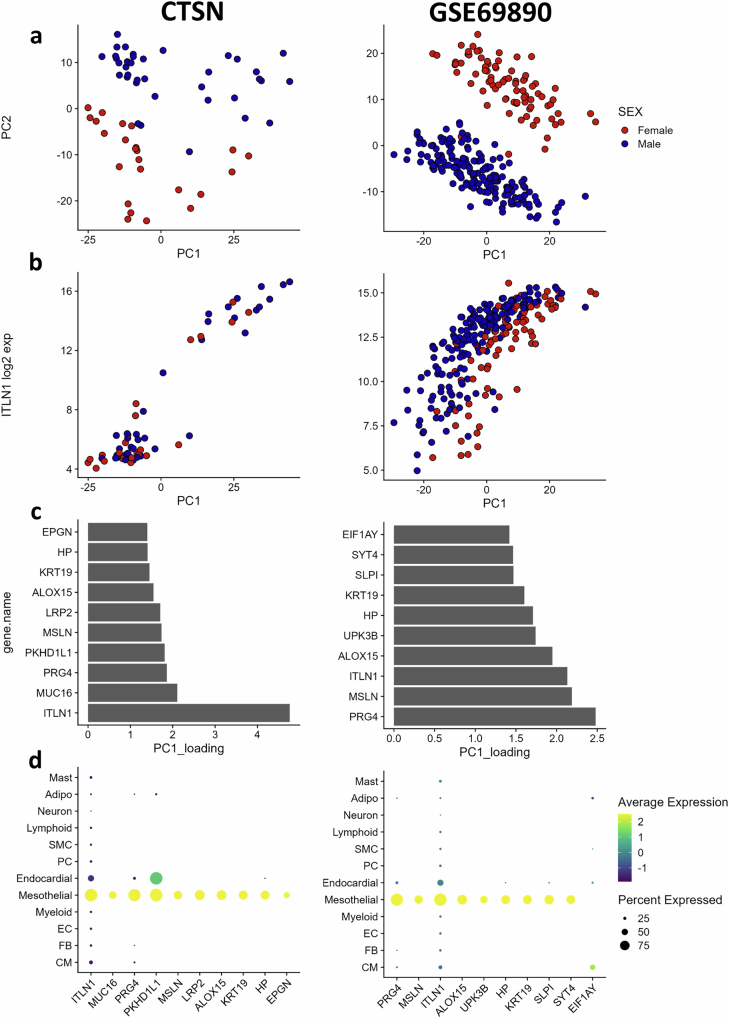
Characterization of two left atrial appendage (LAA) bulk RNAseq dataset. The left and right panels show results from the CTSN cohort (31 AF and 31 SR samples) and GSE69890 (130 AF and 50 SR samples), respectively^17^. (**a**) Principal component analysis (PCA) of the 500 most variable genes in each dataset. (**b**) Scatter plot showing the log_2_-transformed expression of the epicardium marker *ITLN1* against the first principal component (PC1). (**c**) Top ten genes with highest loadings for PC1. (**d**) Dotplot showing average normalized expressions for genes in (**c**) by cell-type in the scAF single-nucleus RNAseq LAA dataset. Adipo; Adipocytes, CM; Cardiomyocytes, EC; Endothelial cells, FB; Fibroblasts, PC; Pericytes, SMC; Smooth muscle cells.

**Extended Data Fig. 2:**
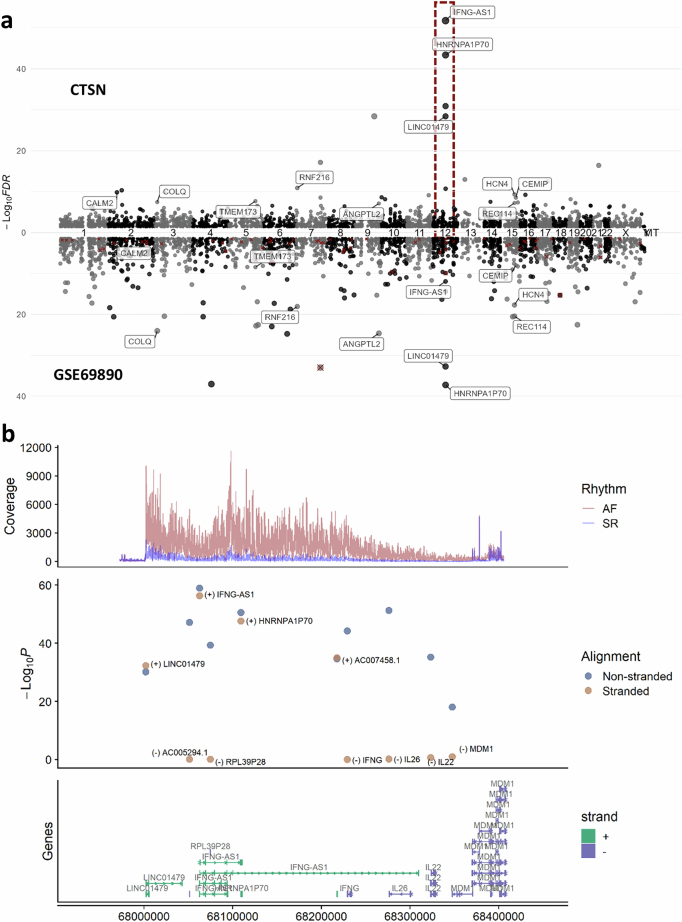
Impact of stand information of differential gene expression in bulk left atrial appendage (LAA) RNAseq datasets. (**a**) Miami plot comparing the bulk RNAseq differential expression between atrial fibrillation (AF) and sinus rhythm (SR) samples -log10(false discovery rates [FDR]) in the CTSN and GSE69890 datasets. Colors and numbers on the x-axis show the chromosomal position of each gene. Red crossed genes denote genes that were only differentially expressed when omitting strand information in the CTSN dataset (Supplementary Table 3). The dashed red box highlights the *IFNG* locus. (**b**) Gene expression profiles at the *IFNG* locus in the CTSN cohort. (top) Coverage plot of all AF and SR samples combined. (center) -log10(FDR) with (orange) and without (blue) strand information provided during read pseudoalignment (Methods). Signs in parentheses show the strand information for each gene. (bottom) Transcript annotation at this locus colored by strand.

**Extended Data Fig. 3:**
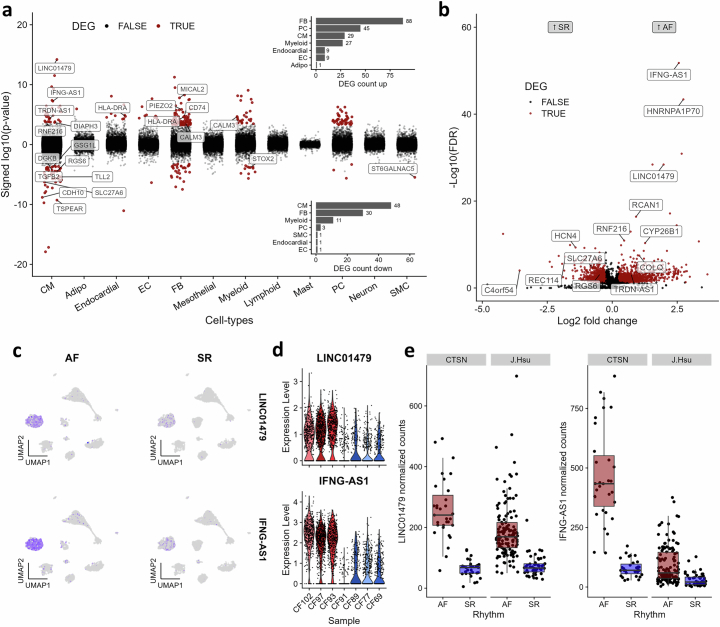
Integration of bulk and single-nucleus RNAseq data in left atrial appendages (LAAs) to identify differentially expressed genes (DEGs) between patients with atrial fibrillation (AF) or control in sinus rhythm (SR). (**a**) Signed log_10_(p-value) for pseudo-bulk differential gene expression analysis of each cell-type between AF and SR samples. The sign is based on the direction of the log_2_ fold-change. Gene colored in red are differentially expressed (false discovery rate [FDR] <0.05). We labeled genes that were also differentially expressed with concordant direction of effect in the two bulk RNAseq datasets. The inset histograms show the number of (top) upregulated and (bottom) downregulated DEGs in each cell-type. (**b**) Volcano plot showing the results of differential expression analysis in the CTSN cohort (LAA bulk RNAseq dataset). Genes colored in red have an FDR < 0.05 and an |log_2_ fold-change | >0.25. (**c**) Single-nucleus uniform manifold approximation and projection (UMAP) colored based on the level of normalized expression of (top) *LINC01479* and (bottom) *IFNG-AS1* in (left) AF and (right) SR patients. This shows increased expression of both genes (blue) in cardiomyocytes of AF patients. (**d**) Violin plots showing the distribution of the cardiomyocytes normalized expression of (top) *LINC01479* and (bottom) *IFNG-AS1* in each LAA sample analyzed in the scAF dataset. Violin distributions of samples colored in red and blue are AF and SR, respectively. (**e**) Boxplots showing the normalized counts of (left) *LINC01479* and (right) *IFNG-AS1* in the two bulk RNAseq cohorts used in this study. J. Hsu corresponds to the NCBI GEO dataset GSE69890. The box plots show the upper quartile, median and lower quartile. Adipo; Adipocytes, CM; Cardiomyocytes, EC; Endothelial cells, FB; Fibroblasts, PC; Pericytes, SMC; Smooth muscle cells.

While some large-scale studies have investigated the changes in gene expression that occur during AF at the whole atrial tissue level^15,16^, an in-depth analysis of the cellular origin of AF DEGs has not been performed. To expand our findings from a small cohort (scAF), we decided to reanalyze two large bulk LAA RNA-seq datasets to identify genes that are robustly differentially expressed between AF and SR. Then we used the scAF data to assign each DEG to the appropriate specific LAA cell type(s). The two bulk LAA RNA-seq datasets totaled 161 persistent AF and 81 SR samples (Methods and Extended Data Fig. 1)^17^. We performed differential gene expression analyses and identified 755 DEGs (false discovery rate (FDR) < 0.05) with concordant direction of effect in both datasets (Supplementary Table 4 and Supplementary Fig. 7). Importantly, we noted that strand information had a dramatic impact on results for the top differentially expressed locus. Specifically, at the *IFNG* locus on chromosome 12, only genes on the positive strand (for example, *LINC01479*, *IFNG-AS1*, *HNRNPA1P70* and *MRPL21* pseudogene) were DEGs between AF and SR when considering strand information, which contrasts with a previous report that implicated genes on the negative strand (for example, *IFNG* and *MDM1*; Extended Data Fig. 2)^15^. In the scAF dataset, we found 118, 77, 48, 38, 10, 10, 1 and 1 DEGs in FBs, CMs, pericytes, myeloid cells, ECs, endocardial cells, adipocytes and smooth muscle cells, respectively (Supplementary Table 4 and Extended Data Fig. 3). When we intersected the LAA bulk and single-nucleus differential expression results, we found 26 DEGs that we could assign to a specific cell type (Supplementary Table 5 and Supplementary Fig. 8). For instance, *LINC01479* and *IFNG-AS1* at the *IFNG* locus were the two strongest DEGs in CMs (Extended Data Fig. 3), and *MICAL2* and *PIEZO2* in FBs.

**Extended Data Fig. 4:**
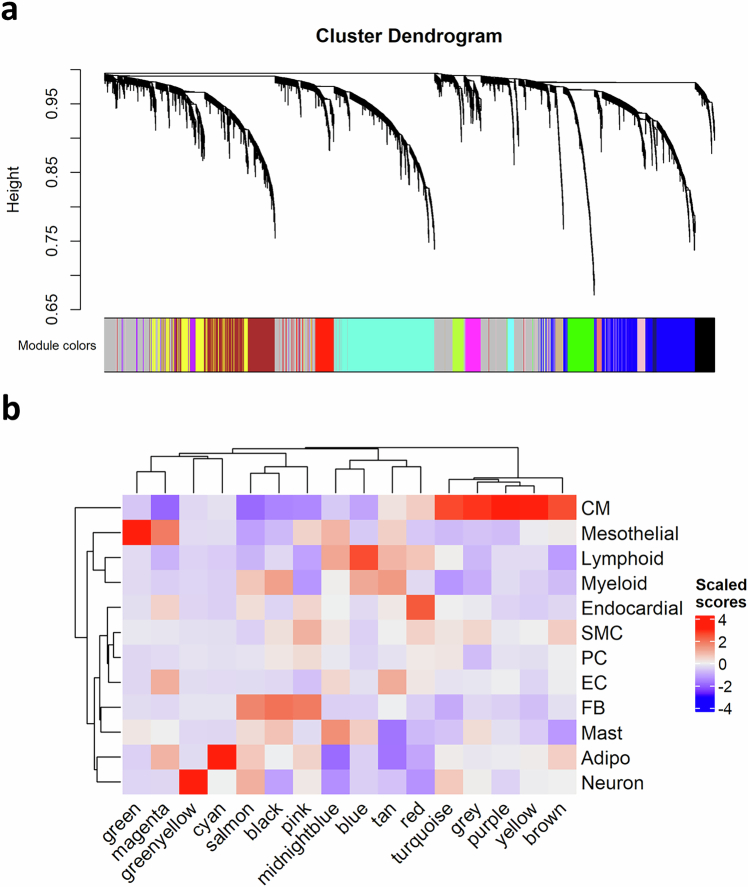
Gene expression module analysis using WGCNA. (**a**) Dendrogram of gene x gene similarity based on their expression in left atrial appendage bulk RNAseq datasets. Each gene’s module attribution by WGCNA is labeled by colors at the bottom. (**b**) Heatmap shows the scaled mean module score (centered and divided by the module standard deviation) by cell-type in the scAF dataset. The dendrograms show similarity in terms of module (x-axis) and cell-type (y-axis). Adipo; Adipocytes, CM; Cardiomyocytes, EC; Endothelial cells, FB; Fibroblasts, PC; Pericytes, SMC; Smooth muscle cells.

**Extended Data Fig. 5:**
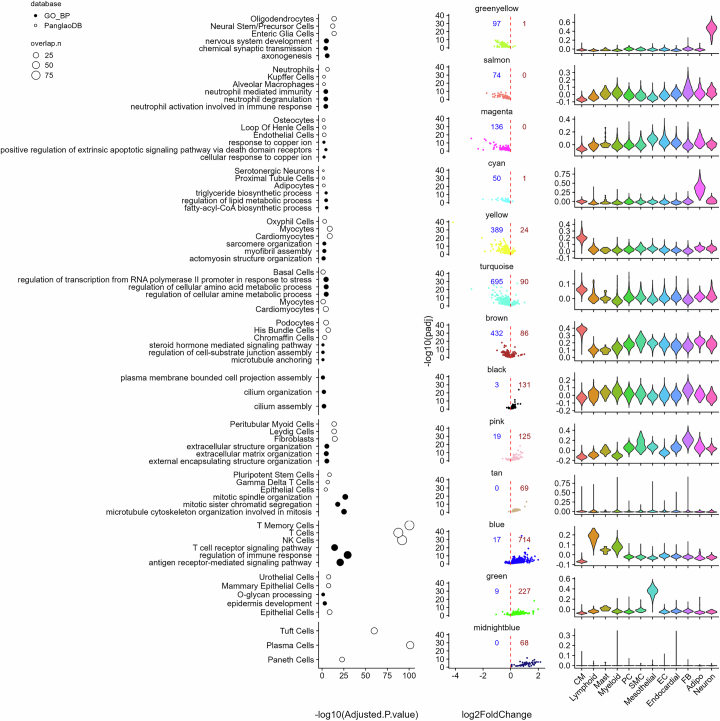
WCGNA bulk RNAseq module gene set overrepresentation analysis. (left) Dot plots that show the top three gene sets from an overrepresentation analysis of the differentially expressed genes (DEGs) in each module. For this analysis, we used the PanglaoDB and gene ontology biological process (GO BP) gene set libraries. (center) Volcano plots that shows the log_2_ fold-change and -log_10_(false discovery rate [FDR]) statistics from the DEG analysis, stratified by module. Red and blue integers indicate the number of atrial fibrillation upregulated and downregulated genes, respectively, in each module. (right) Violin plots showing the distribution of the module scores in each cell-type of the scAF dataset. Adipo; Adipocytes, CM; Cardiomyocytes, EC; Endothelial cells, FB; Fibroblasts, PC; Pericytes, SMC; Smooth muscle cells.

To complement the DEG analyses and try to capture the impact of rarer cell types on AF-associated transcriptional changes, we performed a module analysis using weighted gene coexpression network analysis (WGCNA; Methods). For these analyses, we merged and performed differential gene expression analyses (AF versus SR) on the two bulk RNA-seq datasets together to maximize discovery power. WGCNA partitioned 7,970 genes into 16 modules of variable sizes ranging from 74 to 2,077 genes (Extended Data Fig. 4a). With this approach, we found shared and cell-type-specific modules: for instance, we showed that single-nucleus enrichment scores for the green-yellow and cyan modules (genes mostly downregulated in AF) are highly specific to neurons and adipocytes, respectively, while scores for the blue and green modules (genes mostly upregulated in AF) are highly specific to lymphoid and mesothelial cells, respectively (Extended Data Fig. 4b). In Supplementary Table 6, we annotated each gene in each module with its corresponding differential gene expression result (log_2_ fold change and FDR) from the AF versus SR bulk RNA-seq analyses. We also performed pathway analyses on the genes in each module (Supplementary Table 7). Because for most of these modules, the genes were mostly upregulated (or downregulated) in AF versus SR (volcano plots in Extended Data Fig. 5), our pathway analyses highlighted functions that were activated (or inactivated) in AF versus SR (Extended Data Fig. 5 and Supplementary Table 7).

We can use lymphoid cells to illustrate the added information provided by this module approach. In the scAF dataset, we did not find DEGs between atrial lymphocytes from AF versus SR donors, presumably because lymphocytes only correspond to 1.7% of our single-nucleus dataset. However, in the lymphoid-specific blue WGCNA module, there are 731 genes that are differentially expressed, including 714 that are upregulated in AF (Supplementary Table 6). Pathway analyses with the genes in the WGCNA lymphoid-specific blue module suggest an important contribution by lymphocytes to changes that occur in the general transcriptional landscape of human atria in AF (Discussion). Our module analyses provide more granularity in interpreting our differential gene expression results, allowing us to propose links between the bulk RNA-seq results, rarer cell types and their functions in AF.

### Androgen receptor modulates AF-upregulated genes in LAA CMs

**Fig. 2:**
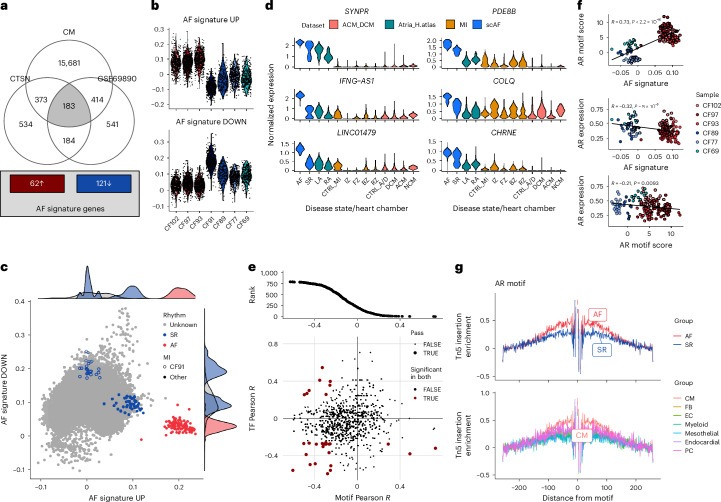
A gene expression signature to identify CMs from individuals with AF. **a**, To create a CM-specific gene expression signature for AF, we identified genes specifically expressed in CMs in the scAF dataset and selected from those genes that are differentially expressed in two large LAA bulk RNA-seq experiments. This prioritized 62 and 121 genes that are upregulated and downregulated, respectively, in AF. **b**, For each scAF participant, we plotted the distribution of the upregulated and downregulated expression signatures in CMs. Blue are SR controls and red are individuals with AF. **c**, We combined CMs from four publicly available cardiac snRNA-seq datasets with scAF. The AF signatures clearly separate CM meta cells from individuals with AF (red) from the rest of the CMs (blue and gray). **d**, Expression of six CM-specific genes from the AF-upregulated signature in various snRNA-seq datasets. ACM, arrhythmogenic cardiomyopathy; ACM_DCM, arrhythmogenic and dilated cardiomyopathy dataset; Atrial_H.Atlas, atrial heart atlas nuclei dataset; BZ, boarder zone; CTRL_A/D, control samples from the ACM_DCM dataset; CTRL_MI, control samples from the MI dataset; DCM, dilated cardiomyopathy; FZ, fibrotic zone; IZ, ischemic zone; LA, left atria; LAA_AF, this study scAF dataset; MI, myocardial infarction dataset; NCM, non-compaction cardiomyopathy; RA, right atria; RZ, remote zone. **e**, The plot at the top shows the correlation coefficients (Pearson’s *r*) between the AF-upregulated signature and TF motif activities in CMs. The scatterplot at the bottom captures the correlation between TF motif and the AF-upregulated signature (*x* axis) and between TF expression level and the AF-upregulated signature in CMs from the scAF dataset (*y* axis). Red points highlight TFs with significant correlations at the level of the motif and expression with the AF-upregulated signature (FDR < 0.01). **f**, In the scAF CMs, the AF-upregulated signature is correlated with the *AR* motif activity (snRNA-seq; top) and with *AR* expression (middle). The *AR* motif and expression are also correlated in CMs (bottom). Blue indicates SR controls and red indicates individuals with AF. For each plot, we calculated Pearson’s *r* and a nominal two-sided *P* value. **g**, The Tn5 footprinting shows a stronger enrichment near AR motifs in CMs versus other cell types, and in CMs from individuals with AF versus SR controls. The results by cell type are shown in Supplementary Fig. 11.

**Extended Data Fig. 6:**
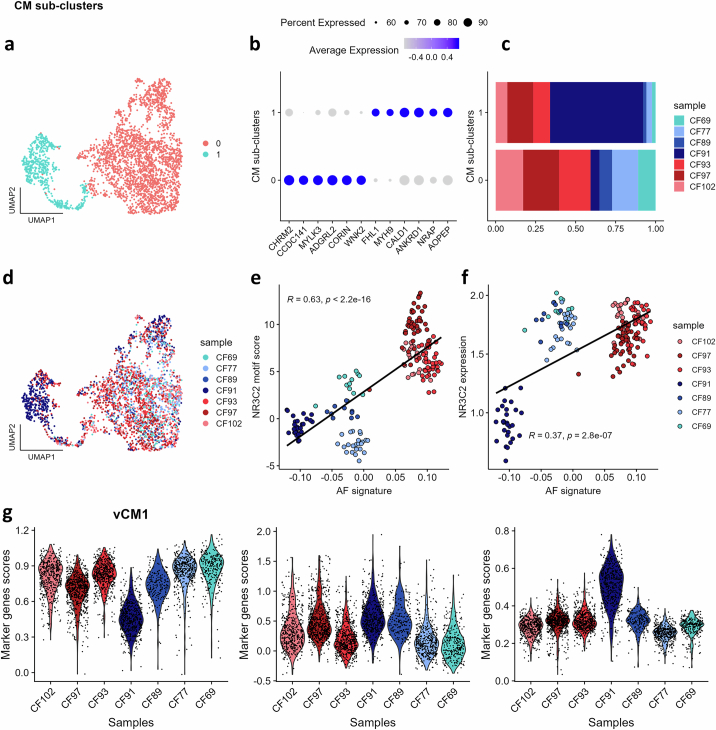
Subcluster analysis of cardiomyocytes (CMs) in the scAF single-nucleus RNAseq dataset did not identify atrial fibrillation (AF)-specific cell clusters, but rather an artifact due to a pre-existing additional heart condition in one of the donors. (**a**) CM uniform manifold approximation and projection (UMAP) colored by the two clusters identified by Seurat. (**b**) Dotplot showing average normalized expressions for the top six marker genes for each CM subcluster in **a**. (**c**) Barplot showing the sample portions in each CM subcluster. Red and blue samples represent AF and sinus rhythm (SR) patients, respectively. (**d**) CM UMAP as in **a** with each donor sample colored differently. **c** and **d** indicate that most of the CMs in subcluster 1 belong to donor CF91. This participant was the only one who suffered from a myocardial infarction less than a year before tissue collection, possibly explaining the strong difference of its CM transcriptome. (**e-f**) Scatter plot of (**e**) NR3C2 motif activity and (**f**) expression correlations with the AF signature UP scores in CM metacells. Red and blue samples represent AF and SR patients respectively. (**g**) Ventricular CM gene scores derived from 3 CM states from normal and myocardial infarction human hearts (Methods) in CM from the scAF dataset.

**Extended Data Fig. 7:**
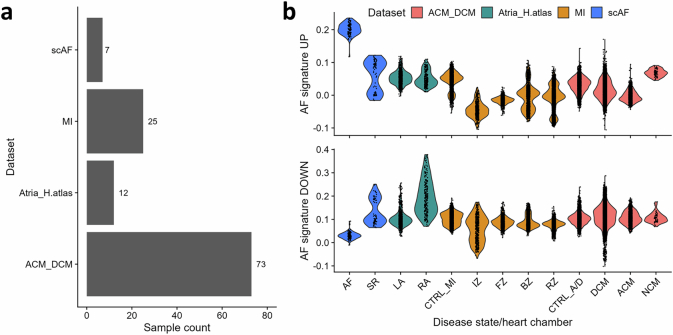
Testing the AF UP and DOWN gene expression signatures in cardiomyocytes (CMs) from independent single-nucleus RNAseq datasets. (**a**) Barplot showing the number of samples found in each dataset used to compare the specificity of the AF signatures. (**b**) Violin plots showing the distribution of the AF signature scores of CM metacells in each disease or cardiac chamber. All comparisons with the scAF AF distribution (leftmost) are significant (*t*-test *P* < 2.2×10^-16^, with the caveat that meta-cells from the same donor are not independent). ACM; arrhythmogenic cardiomyopathy, ACM_DCM; arrhythmogenic and dilated cardiomyopathy dataset, AF; atrial fibrillation, Atrial_H.Atlas; atrial heart atlas nuclei dataset, BZ; boarder zone, CTRL_A/D; control samples from the ACM_DCM dataset, CTRL_MI; control samples from the MI dataset, DCM; dilated cardiomyopathy, FZ; fibrotic zone, IZ; ischemic zone, LA; left atria, LAA_AF; this study scAF dataset, MI; myocardial infarction dataset, NCM; non-compaction cardiomyopathy, RA; right atria, RZ; remote zone, SR; sinus rhythm.

**Extended Data Fig. 8:**
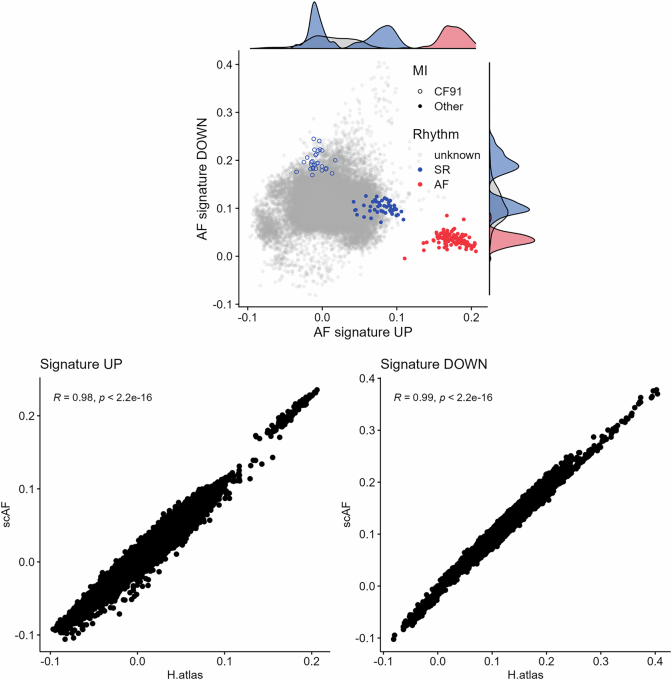
Comparison of the atrial fibrillation (AF) UP and DOWN gene expression signatures when using our scAF and the single-nucleus RNAseq data from the Heart Atlas to select cardiomyocyte (CM)-specific genes. To create the signatures, we intersected genes that are differentially expressed between atrial fibrillation (AF) and sinus rhythm (SR) left atrial appendages in two bulk RNAseq experiments, together with genes that are specifically expressed in CMs as determined by single-nucleus RNAseq using specificity metrics (area under the curve >0.5, Methods). The results presented here show that very concordant AF signatures are obtained when using either our scAF data or the single-nucleus RNAseq data from the Heart Atlas, thus alleviating concerns of data overfitting.

Subclustering analyses of the scAF CM varying multiple parameters (number of principal components, clustering method and outlier inclusion) did not yield an AF-specific cluster (although there was a subcluster likely due to a single SR donor who had a myocardial infarction; Extended Data Fig. 6). As an alternative strategy to identify CM states and possible TFs associated with AF, we derived robust upregulated and downregulated AF gene expression signatures by integrating the bulk and scAF LAA RNA-seq datasets (Fig. 2a and Supplementary Table 8). The AF signatures are sufficient to distinguish between AF and SR samples in CMs of scAF (AF signature upregulated *t*-test *P* value = 0.0017; Fig. 2b). To further test the AF signatures’ specificity to label CMs from individuals with AF, we retrieved external human atrial snRNA-seq datasets (totaling 117 donors) from the Heart Atlas^7^ and two studies focused on specific heart pathologies (dilated cardiomyopathy^8^ and myocardial infarction^18^). The AF transcriptional signatures could discriminate AF CMs from non-AF CMs, including CMs from other heart chambers (Fig. 2c and Extended Data Fig. 7). Importantly, the robust AF signatures are not overfitted to our scAF dataset, as we could generate very similar signatures and results using independent snRNA-seq data from the Heart Atlas (Extended Data Fig. 8). Given the performance of the AF-upregulated signature, we sought to identify its gene members that would represent particularly compelling CM biomarkers (and potential drug targets) in AF by scoring their expression specificity to CMs in AF. Along the long noncoding RNAs *LINC01479* and *IFNG-AS1* (see above), we also found *SYNPR* (synaptoporin), *COLQ* (collagen-like tail subunit of asymmetric acetylcholinesterase), *CHRNE* (cholinergic receptor nicotinic epsilon subunit) and *PDE8B* (phosphodiesterase 8B) as strong candidate genes to validate in future functional experiments (Fig. 2d and Supplementary Table 9).

To prioritize TFs that are important modulators of the CM transcriptome in individuals with AF, we correlated TF binding motif activities (from the snATAC-seq component of the scAF dataset) or TF expression levels (from the snRNA-seq component of the scAF dataset) with the AF-upregulated signature (Fig. 2e and Supplementary Table 10); similar results were obtained with the downregulated signature (Supplementary Fig. 9 and Supplementary Table 10). CMs from the SR donor who had a myocardial infarction appeared as outliers and were excluded from downstream analyses (CF91 in Fig. 2c and Extended Data Fig. 8; see also Extended Data Fig. 6). This analysis identified three genes that share similar TF binding motifs as the strongest signals: the androgen receptor (*AR*) and nuclear receptor subfamily 3 group C members 1 and 2 (*NR3C1* and *NR3C2*; Fig. 2e, Supplementary Fig. 9 and Supplementary Table 10). Of these three genes, *AR* was the only DEG (downregulated) in LAA AF versus SR samples (Supplementary Fig. 10 and Supplementary Table 4), and in scAF, the AF-upregulated signature was correlated with AR binding motif activity and anti-correlated with *AR* expression level (Fig. 2f). We visualized the binding motif activity results using TF footprinting analyses on the scAF ATAC-seq peaks, showing more Tn5 insertions around AR motifs in individuals with AF compared to SR donors, as well as in CMs compared to other cell types (Fig. 2g and Supplementary Fig. 11). These results suggest that androgen signaling and AR activity are key modulators of differential gene expression in CMs from LAAs between individuals with AF and SR controls. This analysis of TF expression and binding motif activity highlighted many other TFs potentially relevant to AF pathophysiology in CMs (without being exclusively expressed in CMs), including *NFIC*, *RORB* and *MXI1* (Fig. 2e, Supplementary Fig. 9 and Supplementary Table 10).

#### Subtypes of FBs in human LAAs

**Fig. 3:**
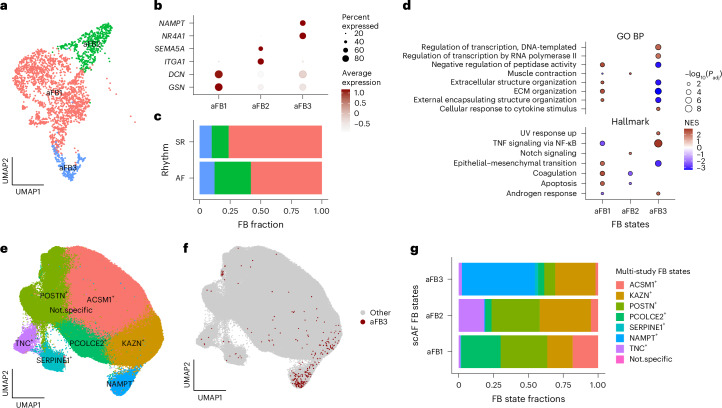
FB subtypes in human LAAs. **a**–**d**, Different analyses of FBs in our scAF multiome dataset. **e**–**g**, Summary of analyses in which the scAF FB was integrated with FBs from additional cardiac single-nucleus datasets. **a**, FB UMAP colored by FB subtype. **b**, Dot plot showing averaged normalized expressions for the strongest marker genes for each FB subtype. **c**, Bar plot showing the FB subtype proportion by rhythm. **d**, Dot plots showing the top three gene sets (by enrichment scores) in each FB subtype from a gene-set overrepresentation analysis based on specificity of expression. For this analysis, we used the Hallmark and Gene Ontology Biological Process (GO BP) gene-set libraries. Gene-set *P* values were adjusted (*P*_adj_) for multiple testing (Benjamini–Hochberg method) below 0.05 (fast gene-set enrichment analysis enrichment *P* values are estimated using an adaptive multilevel split Monte Carlo method). **e**,**f**, Integrated FB UMAP of four human cardiac datasets (Methods). **e**, Eight clusters labeled by their marker genes (the ‘Not.specific’ cluster designates a small proportion of cells for which we could not identify a strong gene marker). **f**, UMAP positions of the aFB3 cluster from the scAF dataset. **g**, Corresponding fractions of the FB subtypes identified in scAF to FB subtypes identified in the multi-study integrated FB clusters in **e**. NES, normalized enrichment score; NF-κB, nuclear factor kappa B.

FBs represent the second-most abundant cell type in LAAs (Fig. 1b) and have a clear role in AF pathophysiology through their fundamental contribution to fibrosis^19^. Our subcluster analysis of FBs in scAF resulted in three FB subclusters: aFB1 (DCN^+^GSN^+^), aFB2 (ITGA1^+^SASH1^+^) and aFB3 (NR4A1^+^NAMPT^+^; Fig. 3a,b and Supplementary Table 11). The proportions of the different FB subtypes between AF and SR LAAs were not significantly different (*P* = 0.057, *P* = 0.4 and *P* = 0.4 for aFB1, aFB2 and aFB3, respectively; Fig. 3c). aFB1 expresses *DCN* and *GSN*, two canonical FB markers^7^, while aFB2 is enriched for fibrotic markers and smooth muscle contraction genes (*TNC*, *COL1A1* and *ACTA2*)^7^ (Supplementary Table 11). Cells in the aFB3 cluster express *NR4A1*, *THBS1* and *CCL2*, and have been less characterized in the literature. To gain better insights into the potential different functions of these FB subtypes, we performed pathway analyses using genes specifically expressed in each of the scAF FB subclusters (Fig. 3d). We found an enrichment of genes involved in Notch signaling and muscle contraction in aFB2, suggestive of a myofibroblast phenotype^20^. Cells in the aFB1 and aFB3 clusters were characterized by contrasting pathways. For instance, aFB3 had a depletion of genes involved in ECM reorganization and an enrichment of genes implicated in inflammatory response (via tumor necrosis factor/nuclear factor kappa B), whereas genes in the same pathways were enriched and depleted, respectively, in aFB1 (Fig. 3d). Because they have been less studied and present a transcriptional profile that distinguish them from aFB1 clusters (which express canonical FB marker genes), we decided to further investigate cells in the aFB3 cluster.

**Extended Data Fig. 9:**
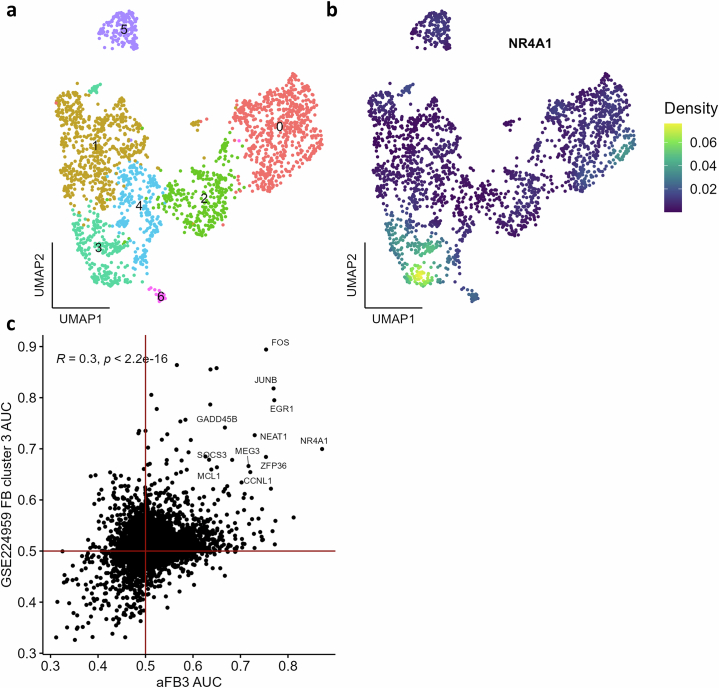
Validation of aFB3 in external single-nucleus RNAseq of left atrial appendages obtained from patients with AF or in normal SR. (**a-b**) Uniform manifold approximation and projection (UMAP) of the fibroblasts (FB) in the GSE224959 single-nucleus RNAseq dataset^23^, showing (**a**) FB subtype and (**b**) *NR4A1* density of expression. (**c**) Scatter plot of gene specificity of expression measured by area under the receiving operating curve (AUC) for aFB3 of the scAF dataset and cluster 3 in (**a**). The Pearson’s p-value and correlation coefficient are shown.

To validate our observation that at least three FB subtypes are present in the human heart, we integrated our scAF FBs with three additional single-nucleus human cardiac datasets. In this harmonized dataset, we found eight FB subtypes, which we labeled with the top marker genes of each cluster (Fig. 3e). This analysis not only allowed us to replicate the presence of aFB3-like cells in the atria of individuals with persistent AF from an independent study (Extended Data Fig. 9)^21^, but also identified FBs with concordant marker genes in the four chambers of normal hearts as well as in the ventricle of diseased hearts: the NAMPT^+^ cluster in Fig. 3e includes most aFB3 cells from our scAF dataset (Fig. 3f)^7,8,22^. This is shown explicitly in Fig. 3g, with around 50% of the scAF aFB3 cells mapping to the NAMPT^+^ cluster.

#### *NR4A1* as a marker of aFB3 regulates atrial FB activity

**Fig. 4:**
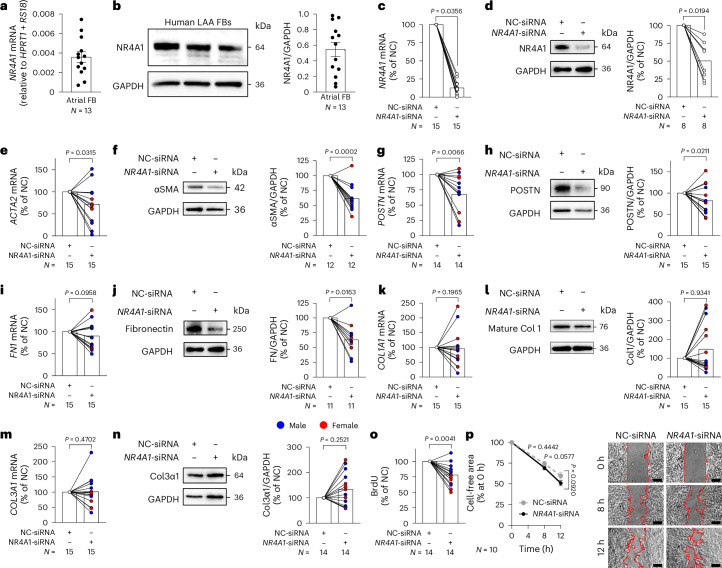
*NR4A1* deficiency reduces expression of fibrotic markers and alters the function of human atrial FBs. **a**, *NR4A1* mRNA levels (quantified by quantitative PCR with reverse transcription (RT–qPCR), relative to the geometric mean of housekeeping genes *HPRT1* and *RS18*) in human atrial FBs. **b**, Representative immunoblots of NR4A1 protein (normalized to GAPDH) in human atrial FBs. **c**,**d**, siRNA-mediated knockdown of *NR4A1* (*NR4A1*-KD) mRNA (analyzed by RT–qPCR) and protein (immunoblotting) in human atrial FBs. **e**–**n**, Effect of *NR4A1*-KD on expression of selected fibrotic markers: αSMA (**e** and **f**), periostin (**g** and **h**), fibronectin (**i** and **j**), type I collagen (Col 1; **k** and **l**) and type III collagen (**m** and **n**) in atrial FBs. **o**, Effects of *NR4A1*-KD on 24-h proliferation of human atrial FBs. **p**, Effects of *NR4A1*-KD on cell migration (scratch assay) at 8-h and 12-h time points. Scale bar, 200 µm. Data are the mean ± s.e.m. (**a**, **b** and **p**) or mean with paired lines (**c**–**o**); each dot (**a** and **b**) or paired line (**c**–**o**) represents an independent biological replicate. Data are expressed as a percentage of the control group (NC-siRNA; **c**–**o**) or the percentage at 0 h (**p**). *P* values were determined from raw values (2^−^^∆Ct^) and reported as two sided using a paired *t*-test (**c**, **d**, **j** and **o**), ratio paired *t*-test (**e**–**i**, **k**, **m** and **n**), Wilcoxon test (**l**) and two-way analysis of variance (ANOVA) with Sidak correction (**p**). *N*, individual biological donors. Source data

One of the most specific markers of aFB3 identified in the scAF FB analysis and confirmed in FBs from an independent single-cell LAA dataset is *NR4A1* (Supplementary Fig. 11 and Supplementary Table 11)^23^. To characterize the potential role of *NR4A1* in atrial fibrogenesis and AF, we performed in vitro mechanistic studies using FBs isolated from LAAs of five individuals with persistent AF and ten control (in SR) donors (Supplementary Table 12) and initially checked the *NR4A1* gene and protein expression in these cells (Fig. 4a,b). To test whether NR4A1 may exert any effects on human atrial FB functions, we compared expression of some prominent fibrotic markers in FBs between *NR4A1* siRNA-mediated knockdown (*NR4A1*-KD) and negative non-targeting siRNA-control (NC-siRNA; Fig. 4c,d). We found that mRNA and protein expression of α-smooth muscle actin (αSMA, encoded by *ACTA2* gene), a marker of the fibroblast-to-myofibroblast activation, was lower in *NR4A1*-KD atrial FBs compared to control cells transfected with NC-siRNA (Fig. 4e,f). In addition to FB activation, ECM accumulation is a major component of fibrosis^19^, which is characterized by increased production of key ECM components, such as type I collagen (*COL1A1*), type III collagen (*COL3A1*), fibronectin (*FN1*) and periostin (*POSTN*). The *NR4A1*-KD decreased *POSTN* transcript and protein (Fig. 4g,h), as well as fibronectin protein (*P* = 0.0163) and showed a trend toward a reduction in fibronectin mRNA expression (*P* = 0.0958; Fig. 4i,j). By contrast, type I collagen and type III collagen mRNA and protein expression remained unaltered in *NR4A1-*KD human atrial FBs (Fig. 4k–n). These results point toward the NR4A1 regulatory role in key fibrotic responses in human LAA FBs by promoting FB activation and production of the major ECM components.

FB proliferation and migration are important contributors to fibrogenesis. We found that human atrial FBs deficient in *NR4A1* proliferated more slowly compared to the NC-siRNA cells (Fig. 4o). Furthermore, *NR4A1*-KD cells showed a trend toward an increase in atrial FB migration (Fig. 4p). Thus, in addition to the activation of some important profibrotic responses, *NR4A1* also promotes proliferation of atrial FBs.

**Extended Data Fig. 10:**
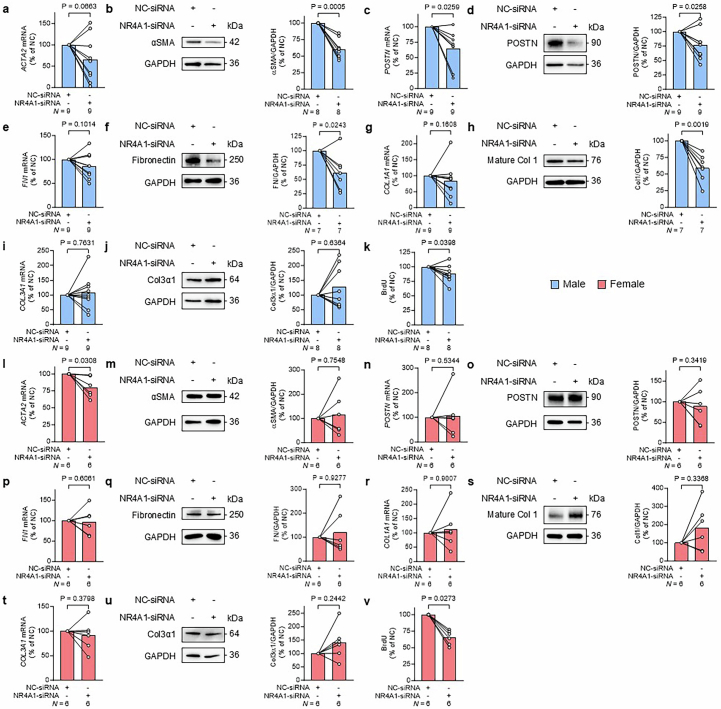
Sub-analysis showed sex-specific differences in NR4A1 regulation of fibrotic responses in human atrial fibroblasts (FBs). (**a-k**) Effect of *NR4A1* siRNA-mediated knockdown (*NR4A1*-KD) on expression of selected fibrotic markers: αSMA (**a-b**), periostin (**c-d**), fibronectin (**e-f**), collagen 1 (**g-h**) and collagen 3 (**i-j**) in male atrial FBs (blue bars). (**k**) Effects of *NR4A1*-KD on 24-hour proliferation of male FBs. (**l-v**) Effect of *NR4A1*-KD on expression of selected fibrotic markers: αSMA (**l-m**), periostin (**n-o**), fibronectin (**p-q**), collagen 1 (**r-s**) and collagen 3 (**t-u**) in female atrial FBs (red bars). (**v**) Effects of *NR4A1*-KD on 24-hour proliferation of female FBs. Data are mean with paired lines; each paired line represents an independent biological replicate. Expression is expressed as % of control group (NC-siRNA). *P* values were determined from raw values (2^-∆Ct^) using ratio paired t-test (**a-g**, **i-j**, **l-u**) and paired t-test (**h**, **k**, **v**). *N*, individual biological donors. Source data

As persistent AF is characterized by sex-specific differences for clinical prevalence and outcomes^24^, we tested whether the NR4A1 regulation of atrial FB functions might differ in female and male donors. A sub-analysis of the above findings separately in male and female atrial FBs found that male *NR4A1*-KD atrial FBs had a lower expression of αSMA protein (*P* = 0.0005) and a trend toward a decrease in its mRNA (*P* = 0.0663) (Extended Data Fig. 10a,b), as well as decreased periostin mRNA and protein expression (Extended Data Fig. 10c,d). Furthermore, the *NR4A1*-KD caused a reduction in fibronectin and type I collagen protein levels with unaltered transcript expression in male FBs (Extended Data Fig. 10e–h. The type III collagen mRNA and protein remained unchanged in NR4A1-deficient male FBs (Extended Data Fig. 10i,j), while cell proliferation was significantly slower in these cells (Extended Data Fig. 10k).

In female atrial FBs, the *NR4A1*-KD was accompanied by a decrease in mRNA (without changes in protein) expression of αSMA (Extended Data Fig. 10l,m). Moreover, unlike in male FBs, *NR4A1*-KD did not alter the expression of other profibrotic markers (periostin, fibronectin and type I and type III collagen) in female FBs (Extended Data Fig. 10n–u). However, the *NR4A1*-KD female cells had a slower cell proliferation (Extended Data Fig. 10v). Collectively, these results demonstrate that NR4A1-mediated effects are more pronounced in male atrial FBs; thus, this study provides insights on the potential role of sex-associated differences in the NR4A1-mediated responses in human atrial FBs.

## Discussion

Transcriptomic and functional analyses allowed us to characterize genes, expression modules and cell populations implicated in human AF. To maximize the robustness of our results, (1) we integrated results from two large bulk RNA-seq experiments with a unique AF single-nucleus multiome dataset, (2) we only analyzed LAAs because the left atrium is the predominant heart chamber affected by AF^25^, and (3) we only collected diseased biospecimens from individuals with persistent AF. Our results provide transcriptomic and mechanistic insights into some of the triggers responsible for long-term structural remodeling and perpetuation of arrhythmogenic substrates in the heart.

We identified multiple genes that are robustly differentially expressed in AF, including genes with known roles in CMs (*HCN4*, *RCAN1*, *CALM3* and *RGS6*) as well as those that have been less studied in this cell type (*ANGPTL2*, *REC114*, *RNF216*, *C4orf54*, *LINC01479* and *IFNG-AS1*). While we identified these genes as most likely differentially expressed in bulk RNA-seq due to changes in CM transcription during AF, they are not necessarily expressed only in CM. For instance, *IFNG-AS1* is generally associated with immune cell *IFNG* regulation^26^. Using the DEG list, we built an expression signature that can identify AF CMs specifically. Taking advantage of our snATAC-seq, we showed that AR binding motifs are enriched in the regulatory regions of the genes in this signature. There is evidence linking AR signaling with AF, for example: (1) low dihydrotestosterone has been reported to increase AF risk in older men^27^; (2) during hormone cancer therapies, androgen deprivation therapy was associated with increased QT interval duration^28^; and (3) AR knockout mice have impaired Ca^2+^ homeostasis^29^.

To uncover genes that are expressed in rarer cell types but potentially also important for AF, we aggregated co-regulated genes into modules. This approach suggested increased immune cell proportions or activities in AF. Specifically, T cells appeared to best explain one of the immune gene modules enriched for upregulated AF DEGs (blue module). A small module showed strong enrichment in B-cell-specific genes, but we did not detect B cells in our scAF dataset. Both T cells and B cells have been reported to be more prevalent (although B cells are rarely observed) in LAAs of individuals with AF compared to SR^30^. We found two modules composed almost exclusively of downregulated DEGs in AF that are specific to either neuronal cells or adipocytes, suggesting that these cell types may be depleted in individuals with AF. While increased pericardial adipose tissue content has been associated with AF^31^, AF progression has been associated with a progressive loss of sub-epicardial adipocytes, which anti-correlated with sub-epicardial fibrosis^32^.

Our transcriptomic approaches highlighted other interesting AF gene targets that show high disease and/or cell-type specificity, including *PDE8B*, *COLQ*, *CHRNE* and *SYNPR*. Supporting our approach, *PDE8B* (phosphodiesterase 8B) has recently been shown to alter l-type calcium current in individuals with AF^33^. Phosphodiesterases hydrolyze the cyclic second messengers cAMP and cGMP, directly impacting a host of CM functions such as contractility, stress response and gene transcription^34^. Moreover, CMs have age-dependent intrinsic acetylcholine (ACh) synthesis, storage and transport properties^35^, which, when altered, impact CM size^36^. *COLQ* is one of the most replicated DEGs in AF^6^. It anchors acetylcholinesterase (which hydrolyzes ACh) at neuromuscular junctions^37^. *CHRNE* encodes an ACh receptor (AChR) subunit also found at neuromuscular junctions^37^ and is enriched in the atria^38^. Mutations in this gene are associated with congenital myasthenic syndrome, causing postsynaptic Ca^2+^ overload^39^. The upregulation of *COLQ* and *CHRNE* would lead to increased ACh signaling, the former through increased extracellular acetylcholinesterase anchoring and the latter through increased AChR formation. Lastly, *SYNPR* encodes a synaptic vesicular membrane component and is one of the most atrial CM-specific genes in the Heart Atlas^7^, although its role in the heart remains to be determined.

Our subclustering analysis of LAA FBs identified three subclusters, one of which—aFB3—has high *NR4A1* and *NAMPT* expression compared to other FBs and has been less studied. Transcriptional clustering of FBs isolated from human right atria also identified a FB subtype with concordant marker genes to aFB3 (ref. ^21^). In individuals with cardiomyopathy, ventricular FBs with an aFB3-like transcriptome were found to be less abundant, an observation that prompted the authors to assign a cardioprotective role to these cells^8^. The role of *NR4A1*, the top marker of aFB3, in cardiac fibrosis remains unclear. Emerging evidence implicates *NR4A1* in FB differentiation, proliferation and migration^40^, calcium handling and apoptosis of CMs^41–43^, the endothelial-to-mesenchymal transition^44^ and cardiac pathophysiology, including fibrosis^40,45,46^. It has been suggested that *NR4A1* may exert anti-fibrotic effects after myocardial infarction^44^, although findings from rat neonatal atrial FBs show that NR4A1 exerts cellular profibrotic effects^40^. It remains unknown whether the same outcomes can be replicated in mature human atrial FBs. Our study found that NR4A1 regulates some key fibrotic responses in native human atrial FBs. Specifically, the siRNA-mediated *NR4A1*-KD suppressed synthesis of key profibrotic markers and proliferation of human atrial FBs. Although NR4A1 has been previously shown to regulate fibrosis in a transforming growth factor (TGF)-β-dependent manner^40,47^, we observed that in human FBs, the *NR4A1* is capable of altering fibrotic responses even in the absence of TGF-β stimulation. Understanding the downstream signaling of NR4A1 in these cells may uncover mechanistic targets in atrial fibrogenesis.

Previous work reports sex differences in atrial structural remodeling in individuals with persistent AF^48^. Females display a greater extent of fibrosis and upregulation of targets involved in the TGF-β–Smad3 signaling pathway. These observations may provide a potential explanation of the higher risk of AF recurrence in women than in men after ablation or pharmacological treatments^49^. By the same token, we noted that female and male atrial FBs respond differently to the *NR4A1*-KD cells, with male cells exerting more pronounced effects compared to female cells. These results point toward some sex-dependent differences in NR4A1-mediated functions in atrial FBs as potentially important contributors to atrial fibrogenesis.

Similar effects have been previously reported in white adipose tissue and the setting of metabolic homeostasis^50^. NR4A1 belongs to the steroid and thyroid hormone and retinoid receptor superfamily, and it has been suggested that differences in endogenous testosterone and estrogens regulate NR4A1 expression in males and females, respectively^50^. In cardiac FBs, estrogen-mediated signaling inhibits the proliferation and collagen production. Therefore, physiological differences between males and females, including the pleiotropic effects of the endocrine signaling, might contribute to the distinct effects of NR4A1 in male and female atrial FBs. The sex differences in NR4A1 regulation can be followed up in a more comprehensive study.

Our study has several limitations. First, most modules could be confidently attributed to a specific cell type based on concordant pathway and single-cell enrichments. However, other cell types that were not detected in our scAF dataset (such as plasma cells in the midnight-blue module) may also explain the co-regulation of genes observed in bulk RNA-seq data. Integration of our current transcriptomic datasets with proteomic analysis may provide an explanation for these differences^7^. Second, the limited sample size of the scAF dataset is likely to preclude the identification of modestly dysregulated genes involved in AF pathogenesis. We tried to address this issue by taking advantage of the large bulk RNA-seq datasets to identify transcriptomic modules (WGCNA) and then using our scAF to assign these modules to specific cell types. The genes and pathways prioritized in rare cell types by this approach are promising but need to be externally validated. In the future, larger-scale (to minimize participant-specific biases) single-cell datasets in individuals with AF could be especially informative to identify DEGs and cell-state transitions in rare cell types such as neuronal cells and adipocytes. Third, our selection of TFs is partly based on their transcriptional level, which does not account for posttranslational regulation mechanisms. Therefore, motif enrichments due to nuclear translocation events can be missed. Fourth, the culture conditions unavoidably promote FB transition into activated FB phenotypes (myofibroblasts) and cannot fully integrate the complex cell–cell molecular interactions in physiological environments. Thus, these results warrant future in vivo studies in clinically relevant mouse models of persistent AF. Fifth, the comparisons between our scAF dataset and other single-nucleus human heart datasets could be affected by confounding factors such as the tissue of origin or the quality-control metrics used. While we used a state-of-the-art method to integrate these different datasets, we cannot rule out slight biases in the interpretation of our results due to these factors. Finally, our functional characterization focused on a single gene, *NR4A1*, which is unlikely to account for all functions in the aFB3 subtype. Future studies could target other enriched genes in aFB3, such as *NAMPT*.

In this study, we profiled a multiome dataset of human LAAs from individuals with persistent AF and controls in SR and integrated its results with LAA bulk RNA-seq to identify genes, TFs, networks/modules and transcriptomic signatures that are relevant for AF. Importantly, using the single-nucleus modality, we could prioritize in which cell-type genes these elements are differentially expressed and may, therefore, contribute to AF development or progression. Our mechanistic studies focused on *NR4A1*, a gene that marks a poorly characterized subpopulation of atrial FBs. Collectively, our study provides valuable insights into human AF pathophysiology, including a list of genes that represent strong targets to guide the development of therapeutics for AF.

## Methods

### Human cohorts and sample preparation

Studies in human heart tissue and FBs have been approved by the South Central-Berkshire B Research Ethics Committee (UK, ref. 18/SC/0404). All participants gave informed consent before enrollment. Studies include individuals scheduled for an elective cardiac surgery (valve repair/replacement or coronary artery bypass grafting; Supplementary Tables 1 and 12) at the John Radcliffe Hospital, Oxford. LAA biopsy samples were collected and processed for snRNA-seq or FB isolation as described below.

The individuals included for sn-multiome were consecutively recruited to the study and classified, in the first instance, by heart rhythm (AF versus controls in SR). The AF group was defined as individuals with an electrocardiogram-evidenced diagnosis of persistent AF. Controls were identified as individuals with no history of AF or other heart rhythm disturbance and who remained in SR for a 4-day period of postoperative follow-up. Both sexes were included. Individuals aged over 85 years, those who are younger than 18 years or those with a history of previous cardiac surgery were excluded. Moreover, to eliminate confounding conditions, exclusion criteria were individuals with impaired ejection fraction (<50%), chronic kidney disease (estimated glomerular filtration rate < 70 ml min^−1^/1.73 m^2^ or any signs of kidney disease), anemia, type I or II diabetes and history of coronavirus disease 2019 infection within the last 3 months.

### Multiome raw data processing and preprocessing steps

Single-nucleus sample preparation and sequencing, as well as the bioinformatic analyses of the 10x multiome data (alignment and preprocessing) have been described previously^51^. Briefly, we followed the 10x multiome isolation and library construction protocols to prepare the samples (https://www.10xgenomics.com/support/single-cell-multiome-atac-plus-gene-expression/documentation/steps/sample-prep/nuclei-isolation-from-complex-tissues-for-single-cell-multiome-atac-plus-gene-expression-sequencing/). We sequenced the libraries at the Genome Quebec’s Centre d’Excellence et de Services on a NovaSeq 6000 S4 PE100, targeting 30,000 and 60,000 paired reads for the RNA and ATAC libraries, respectively. We aligned the FASTQ data on the GRCh38-2020-A reference using Cell Ranger. We carried out downstream bioinformatic analyses with Seurat (version 4), Signac and Harmony. We removed doublets using scDblFinder and manual curation.

### Subclustering analyses

For both FB and CM subclustering, ten principal components were used from the snRNA-seq modality with the FindNeighbors() and FindClusters() functions. The same remaining processing steps used for the whole dataset were otherwise applied^51^. To identify FB states across cardiac chambers and diseases, we integrated our scAF FBs with three other publicly available datasets^7,8,22^. We used the ‘Harvard-Nuclei’ FBs from the Heart Atlas^7^ (https://cellgeni.cog.sanger.ac.uk/heartcellatlas/data/hca_heart_fibroblasts_raw.h5ad). For ref. ^8^, we downloaded the FB data (https://cellxgene.cziscience.com/collections/e75342a8-0f3b-4ec5-8ee1-245a23e0f7cb/private/) and used the ‘10x 3' v3’ nuclei. For ref. ^22^, we downloaded all nuclei (https://singlecell.broadinstitute.org/single_cell/study/SCP1303/) and selected FBs from the metadata (‘Activated_fibroblast’, ‘Fibroblast_II’, ‘Fibroblast_I’). For each dataset, we performed an independent clustering and removed likely doublets based on marker genes from other cell types. Then, we combined datasets and performed integration by sample using Harmony with ten principal components as described above.

To validate the aFB3 FB state, we downloaded the Gene Expression Omnibus (GEO) dataset GSE224959 (ref. ^23^) human samples, preprocessed data, called doublets and clustered cells as described above. Then, we subclustered FBs, using the same strategy described in this section, and compared area under the curve (AUC) values for scAF aFB3 and GSE224959 FB cluster 3 (Extended Data Fig. 9) using the presto package function wilcoxauc.Seurat().

### ATAC-seq peak comparison with ENCODE and human enhancer atlas

We applied the findOverlaps() function from the R package GenomicRanges to analyze the LAA ATAC-seq peaks in relation to other annotations. Labeling of peaks was completed when they overlapped with other genomic annotations. Subsequently, we utilized the output of Signac’s CallPeaks() function from the cell-type-specific peak calling step to match the LAA ATAC-seq peaks with the corresponding cell type. ENCODE hg38 cCREs (track named encodeCcreCombined) were retrieved from the UCSC Genome Browser. We selected cell-type-specific peaks when peaks were identified in only one cell type. cCREs were retrieved from the human enhancer atlas^52^ and GRanges objects were generated from the downloaded files in http://catlas.org/catlas_downloads/humantissues/cCRE_by_cell_type/. We quantified the percentages of LAA cell-type-specific ATAC-seq peaks overlapping cCREs from each human enhancer atlas cell types. We further applied a 25% threshold and removed scAF-human enhancer atlas cell-type pairs with peak overlaps below the threshold.

### TF prioritization based on cell-type specificity

We used chromVAR^53^ (in Signac^54^ with RunChromVAR() and JASPAR2020; ref. ^11^) to score TF motif AUC in each nucleus. Then, we used the presto package (wilcoxauc.Seurat()) to calculate the AUC for each gene in each cell type. We determined the final TF rank by multiplying the gene and motif AUC.

### CTSN bulk RNA-seq preparation, sequencing and data processing

The preparation of the RNA-seq libraries (including RNA isolation), their sequencing and the data processing steps (including differential gene expression analyses) have been described elsewhere^51^.

#### RNA isolation

The samples were processed with the Bullet Blender Storm method using Green RINO Lysis tubes and 200 µl of QIAzol was added per tube for ~50 mg of tissue, following the manufacturer’s protocol for heart tissue. The RNA was extracted using the miRNeasy Mini Kit (217004, Qiagen), per the manufacturer’s protocol.

### Comparison of bulk RNA-seq DEGs in LAAs from SR and AF donors

The bulk RNA-seq LAA dataset was retrieved from a previous study (GSE69890) with 180 individuals in total (130 with AF and 50 in SR before surgery)^17^. This RNA-seq data were tested using DESeq2 with the same approach as outlined above for the CTSN dataset^55^. PCA was performed to compare PCA between the two RNA-seq datasets and to identify the top 500 variable genes and the influence of sex. Variance stabilizing transformation (DESeq2 function vst()) values were used. The R package ‘factoextra’ was used to retrieve the top genes with the greatest contribution to PC1, followed by checking their expression levels in each cell type from our scAF dataset. Differential expression analyses were achieved using the DESeq() function and included sex as a covariate. Subsequently, log fold change shrinkage was completed using lfcShrink(). For the GSE69890 dataset, individuals in AF rhythm were compared with non-AF controls^16^. The significance threshold of FDR < 0.05 was applied in both datasets, followed by identifying the overlapping DEGs (upregulated and downregulated) from both datasets together with their corresponding log_10_(FDR). To evaluate the effect of the strand, a second pseudocount alignment was performed using kallisto^56^ omitting the -rf-stranded flag. Genes that lost their significance were highlighted when the strand information detected those genes as probable false positives in the GSE69890 non-stranded dataset.

### Single-nucleus differential expression analysis

Differential expression analysis was conducted in the scAF dataset using pseudobulk. Counts were aggregated for each cell type and sample using Seurat’s^57^ AggregateExpression() function, keeping only genes found in more than 5% of nuclei. The same DESeq2 model used for the bulk RNA-seq analyses (above) was also applied, except for the shrinkage model used which was ‘ashr’^58^ instead of the default model. Robust bulk DEGs (FDR < 0.05 and a consistent effect direction) were compared to each cell-type DEG (FDR < 0.05).

### Creating transcriptomic modules with WGCNA

We applied WGCNA on the CTSN and GSE69890 datasets to create gene transcriptomic modules^59^. We merged the datasets to identify genes differentially expressed in AF (adding a dummy variable as a covariate to control for dataset-specific effects). We applied the following filters: base mean expression > 1, |log_2_ fold change | > 0.05 and nominal *P* value < 0.05. This resulted in a dataset of 7,970 genes and 353 LAA samples. We used limma’s removeBatchEffect() to correct for sex and dataset. In WGCNA, we used blockwiseModules() to define transcriptomic modules with the parameters: power = 9, minModuleSize = 50, reassignThreshold = 0, mergeCutHeight = 0.25, networkType = signed; all other parameters were set to default. For biological pathway enrichment analyses, we used enrichR^60^ on genes with FDR < 5% (GO_Biological_Process_2021 and PanglaoDB_Augmented_2021 libraries). For the same genes, we scored the nuclei with AddModuleScore() implemented in Seurat.

### Combined analyses of TF motif and expression in scAF

We created meta cells for FBs and CMs with the package hdWGCNA (MetacellsByGroups(), defined by donor and cell type with the RNA Harmony reduction to aggregate 30 neighboring nuclei with a maximum of 10 overlapping nuclei per meta cell)^61^. The aggregation step was consistent for the snRNA-seq and snATAC-seq modalites. We used Seurat (AddModuleScore()) to calculate the AF signature in each meta cell, and the psych function corr.test() to correlate the AF signature with gene expression levels or TF binding site activities (with FDR correction). We ran Signac’s Footprint() to confirm TF motifs.

### Developing transcriptomic signatures specific to AF CMs from LAAs

We combined bulk RNA-seq and snRNA-seq (scAF) to create gene expression signatures that could specifically label CMs from individuals with AF. In scAF, we used presto’s function wilcoxauc.Seurat() to filter genes with AUC > 0.5 and FDR < 5%. For CTSN and GSE69890, we identified DEGs with FDR < 0.05 and |log_2_ fold change | > 0.25. We used the intersection of these three sets of results to generate the upregulated and downregulated CM-specific AF signatures. The scores of these CM-specific AF signatures were compared between four datasets: two ventricular CM datasets were retrieved from the CELLxGENE portal^8,18^, and the atrial CM dataset was extracted from the Heart Atlas^7^. Meta cells were established as described above, followed by using the AddModuleScore() function to score the cells for the CM-specific AF signatures. To validate the exclusion of the outlier sample CF91 due to a myocardial infarction, we scored CMs using the AddModuleScore() function and the genes from Supplementary Table 10 in ref. ^18^.

To identify prospective therapeutic targets for AF, gene expression from the four datasets in CM meta cells and the scAF cell types were used to recognize genes with the highest specificity to both CMs and AFs. The AUCs were calculated for (1) all scAF cell types and (2) CM meta cell groups using the presto package. Finally, the product of these two AUCs was applied to rank the genes targets from the upregulated AF signatures.

### Isolation and culture of human atrial FB

Human atrial FB isolation was performed as previously described^21^. In brief, LAA biopsy samples were cut into small pieces (~2 mm^3^), incubated twice in digestion solution (4 mg ml^−1^ collagenase type V and 0.0625% trypsin), followed by centrifugation at 2,000*g* for 10 min. Cell pellets were resuspended in FBM-3 Cardiac FB Growth Medium (CC-4526, Lonza) with 10% FBS and supplements (CC-4525, Lonza). Cells were transferred to the six-well plates and incubated at 37 °C and 5% CO_2_ overnight. The medium was replaced every 2–3 days with fresh complete medium. At 80–90% confluency, FBs were washed with 1× PBS, trypsinized and passaged. To minimize culture-associated effects on cell phenotype, FBs of passages P3–P5 were used for all experiments, as validated previously^21^.

### Transfection of human atrial FBs

For gene silencing of *NR4A1*, human atrial FBs were transfected with 50 nM ON-TARGETplus SMARTPool siRNA-targeting *NR4A1* (L-003426-00-0005, Horizon Discovery) or ON-TARGETplus Non-targeting (NC) siRNA as negative control (NC; D-001810-10-05, Horizon Discovery) using Lipofectamine RNAiMAX Transfection Reagent (13778150, Invitrogen). Lipofectamine and siRNA were diluted in FBM-3 antibiotic-free medium with 0.5% FB buffer. At 24 h after transfection, cells were recovered with 10% FBS-containing medium for a further 24 h at 37 °C. Knockdown efficiency was validated by real-time RT–qPCR and western blot to check for reduced gene and protein expression levels, respectively.

### RT–qPCR

Human atrial FBs were lysed in lysis buffer, followed by total RNA isolation (mirVana Kit, AM1561, Invitrogen) according to the manufacturer’s instruction. RNA concentration was measured using the LVis Plate (BMG Labtech). cDNA was prepared by reverse transcription of 100 ng RNA using the QuantiTect Reverse Transcription Kit (205313, Qiagen). RT–qPCR was completed using TaqMan Fast Advanced Master Mix (4444557, Applied Biosystems) and TaqMan Assays (Supplementary Table 13) for specific targets. Each qPCR reaction was run in duplicates on the QuantStudio 7 Flex Real-Time PCR System (Applied Biosystems). Gene expression was quantified using the 2^-∆Ct^ method^62^ and normalized to housekeeping genes (specified in the figures, or figure legends).

### Western blot in human atrial FBs

Human atrial FBs were washed with 1× PBS and lysed in CelLytic M reagent (C2978, Sigma-Aldrich). Protein concentration was measured by Pierce BCA Protein Assay Kit (23227, Thermo Fisher). Cell homogenates were diluted in lysis buffer to obtain the same amount of protein for all samples. Each sample was mixed with NuPAGE LDS Sample Buffer and NuPAGE Sample Reducing Agent, followed by heating at 95 °C for 5 min. Proteins were separated using 4–12% NuPAGE Bis-Tris gel with 1× NuPAGE MES SDS Running Buffer (all from Invitrogen) at constant 170 V for 1 h. Proteins were then transferred with 1× NuPAGE Transfer Buffer (Invitrogen) to polyvinyl difluoride (PVDF) 0.2-µm membranes at 230 A for 1.5 h. Membranes were blocked with 5% skimmed milk in 1× PBS-T for 45 min and incubated with primary antibodies (Supplementary Table 14) at 4 °C overnight. Subsequently, membranes were washed with 1× PBS-T and incubated with horseradish peroxidase-conjugated secondary antibodies at room temperature for 1 h. The immunodetection of the bands was performed with ChemiDoc Imaging System (Bio-Rad) and analyzed by the Image Lab Software (Bio-Rad). Target and housekeeping proteins were run and detected on the same membrane.

### Cell proliferation assay

Cell proliferation was quantified using the BrdU Cell Proliferation Assay (QIA58, Sigma-Aldrich), per the manufacturer’s protocol. Briefly, transfected cells were plated in 96-well plates in triplicates and cultured in medium with 10% FBS. At 70–80% confluency, cells were incubated with BrdU at 37 °C for 24 h, then fixed with fixative/denaturing solution, and incubated with anti-BrdU antibody for 1 h at room temperature. Subsequently, cells were washed three times with 1× wash buffer, followed by a 30-min incubation with Peroxidase Goat Anti-Mouse IgG horseradish peroxidase conjugate. After washing with wash buffer and distilled water, cells were incubated with substrate solution for 15 min in the dark at room temperature. This reaction was then terminated by the addition of a stop solution. The absorbance was measured at 450 nm.

### Cell migration assay

Using a 35-mm µ-dish with a two-well silicone insert with a defined cell-free gap (80206, ibidi), 100 µl of 5 × 10^3^ human atrial FBs were suspended in 10% FBS-containing medium and seeded into each well. The insert was removed at 95% confluence. The medium was then added to the dish, and images were taken at 0-h and 12-h time points. Changes in the cell-free area were analyzed and quantified using Fiji software with a wound-healing size tool^63^. The tool setting was determined as follows: ‘Variance window radius’ = 5 ‘Threshold value’ = 100, ‘Percentage of saturated pixels’ = 0.400, ‘Set scale global?’ = Yes, ‘The scratch is diagonal??’ = No.

### Statistical analyses

Analyses on multiome scAF data were done in R version 4.2.2. Otherwise, statistical analysis was performed using GraphPad Prism 10.1.2 software. Normal distribution was determined by Shapiro–Wilk test. For two-group comparisons, normally distributed data were analyzed by paired *t*-test or ratio paired *t*-test. The non-parametric two-group comparisons were performed by Wilcoxon test (for paired samples). The multiple-group comparisons were analyzed by two-way repeated-measures ANOVA with Sidak’s test. Correlation was assessed by Pearson’s test. For comparisons between categorical variables, a *χ*^2^ test was used. Outliers were identified using the ROUT test and excluded from analysis. Statistical significance was reached when *P* value < 0.05.

### Reporting summary

Further information on research design is available in the Nature Portfolio Reporting Summary linked to this article.

## Supplementary information

Supplementary Information Supplementary Figs. 1–11.

Reporting Summary

Supplementary Tables 1–14.

## Source data

Source Data Fig. 4 Unprocessed western blots.

Source Data Fig. 4 Statistical source data.

Source Data Extended Data Fig. 10 Unprocessed western blots.

Source Data Extended Data Fig. 10 Statistical source data.

## Data Availability

The sn-multiome data discussed in this publication have been deposited in NCBI’s GEO and are accessible through GEO Series accession number GSE238242. The bulk RNA-seq data for the CTSN cohort cannot be released on the GEO but are available from CTSN. Furthermore, we have released the CTSN bulk RNA-seq gene count matrix and anonymized disease status information at http://www.mhi-humangenetics.org/en/resources/. This information is sufficient to reproduce analyses described in this paper.

## Code availability

The code used to analyze the data and generate the figures is available via GitHub at https://github.com/lebf3/scAF_multiome/.
