# Supplementary material for: Single-nucleus multi-omics implicates androgen receptor signaling in cardiomyocytes and NR4A1 regulation in fibroblasts during atrial fibrillation
Source: Nat Cardiovasc Res. 2025 Mar 25;4(4):433–44. doi: 10.1038/s44161-025-00626-0 (PMC11994452; doi:10.1038/s44161-025-00626-0)
Supplement: Supplementary file 1 — Supplementary Figs. 1–11. [file 44161_2025_626_MOESM1_ESM.pdf]

nature cardiovascular research

Supplementary information https://doi.org/10.1038/s44161-025-00626-0

Single-nucleus multi-omics implicates androgen receptor signaling in cardiomyocytes and NR4A1 regulation in fibroblasts during atrial fibrillation

In the format provided by the authors and unedited

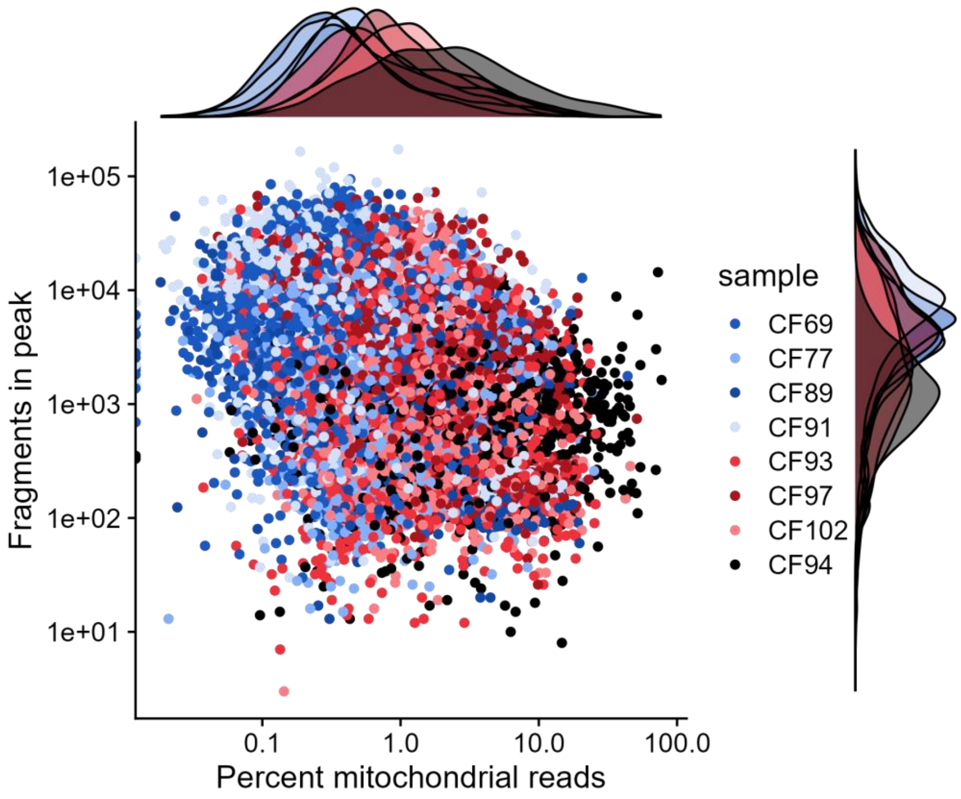

Supplementary Figure 1. Left atrial appendage (LAA) tissue samples used in this multiome study. Scatter plot showing the percentage of mitochondrial read count and number of fragments in peaks. Red and blue dots are from atrial fibrillation and sinus rhythm samples, respectively. We highlight in black the sample (CF94) that was removed based on the poor-quality control metrics.

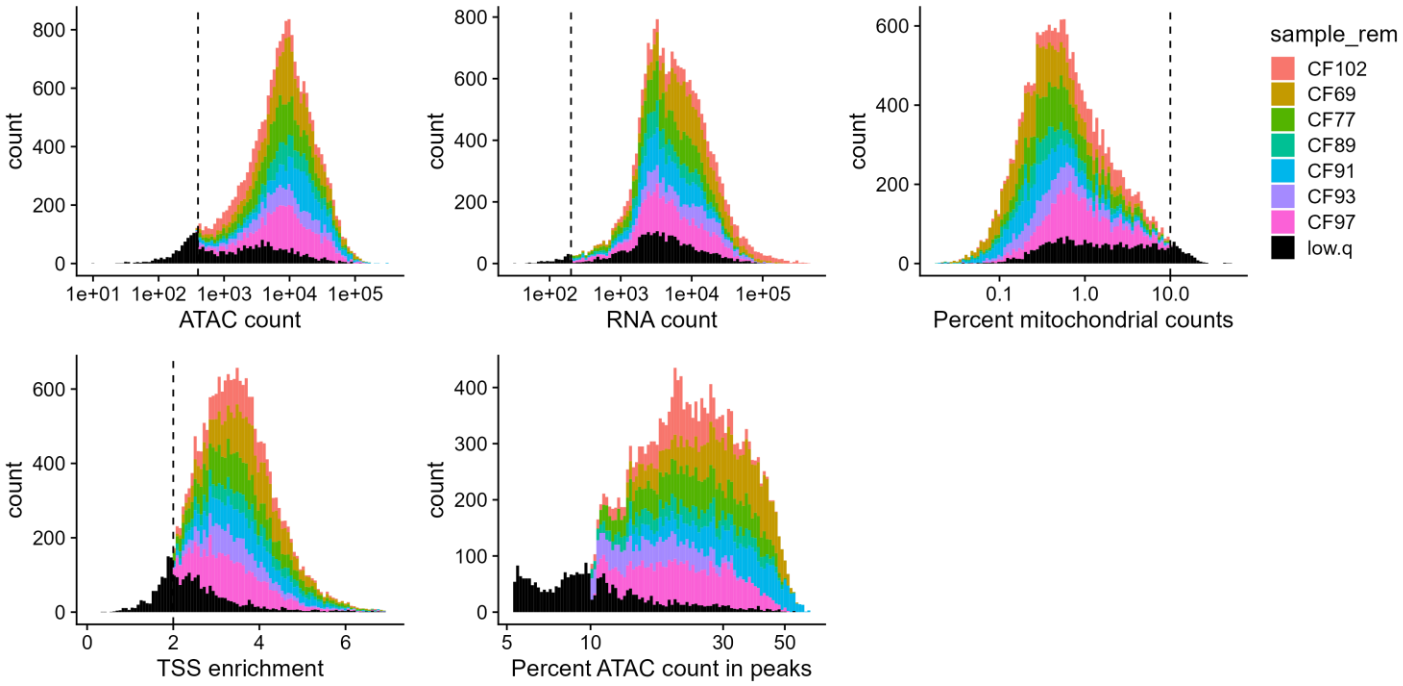

Supplementary Figure 2. Histograms showing thresholds used (dashed vertical lines) for initial filtering of low-quality nuclei. Nuclei in black are the aggregate of nuclei from all samples that do not pass one of the filters. sample_rem; nuclei grouped by sample or binned in the low-quality group, TSS; transcription starting site.

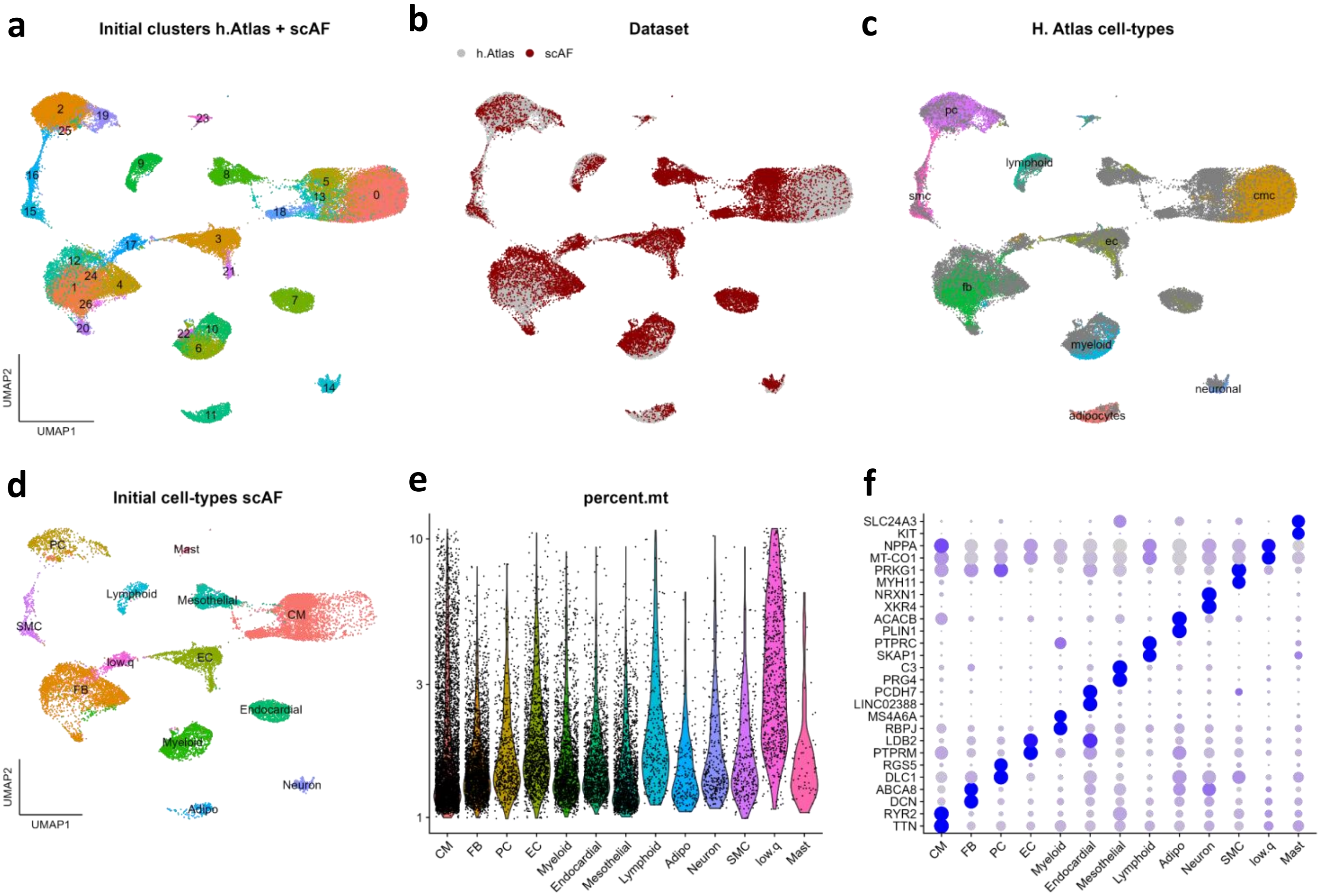

Supplementary Figure 3. Single-nucleus uniform manifold approximation and projection (UMAP) of the integrated nuclei from the heart atlas left atria[7] and the scAF dataset. (**a**) Seurat clusters, (**b**) dataset, (**c**) cell-type labels from the Heart Atlas, and (**d**) Cell-type attributions to the scAF dataset based on their co-clustering with the Heart Atlas labels. (**e**) Violin plot showing the distribution of the percentage of mitochondrial counts in each cell-type of the scAF dataset. (**f**) Dotplot showing average normalized expressions for the top two marker genes for each cell-type in the scAF dataset. pc, pericytes; smc, smooth muscle cells; fb/FB, fibroblasts; cmc/CM, cardiomyocytes; ec/EC, endothelial cells.

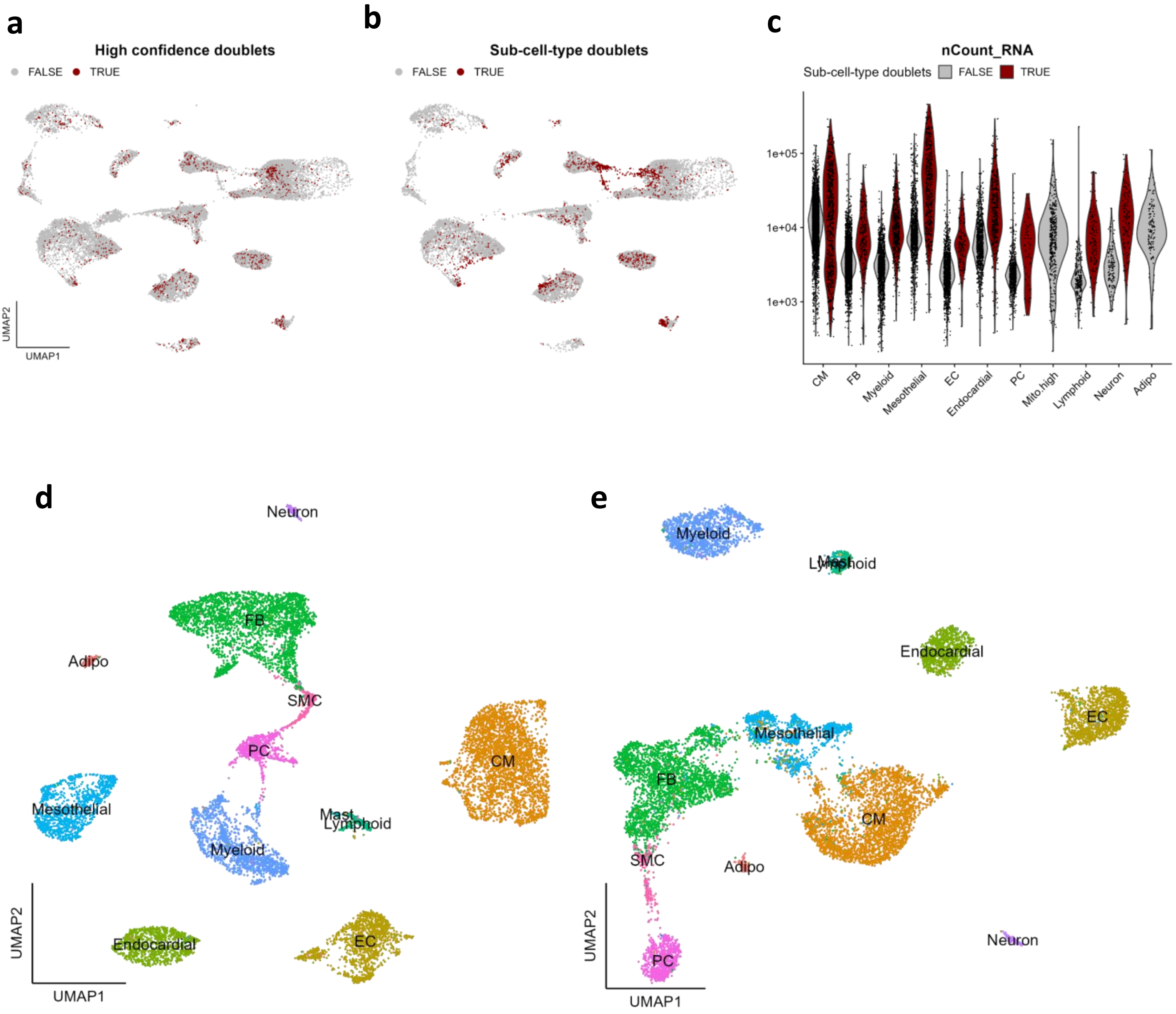

Supplementary Figure 4. Single-nucleus multiome dataset (scAF) uniform manifold approximation and projection (UMAP). (**A**) doublet labels attributed by scDblFinder and (**B**) additional doublets manually attributed based on cell-type markers and scDblFinder scores during subclustering analyses. (**C**) Violin plot showing the distribution of the total RNA read counts per nucleus in each cell-type for doublets and singlets labeled in **B**. Single nuclei UMAP colored by cell-type using the (**D**) RNAseq read count matrix and (**E**) ATACseq fragment count matrix. Adipo; Adipocytes, CM; Cardiomyocytes, EC; Endothelial cells, FB; Fibroblasts, PC; Pericytes, SMC; Smooth muscle cells.

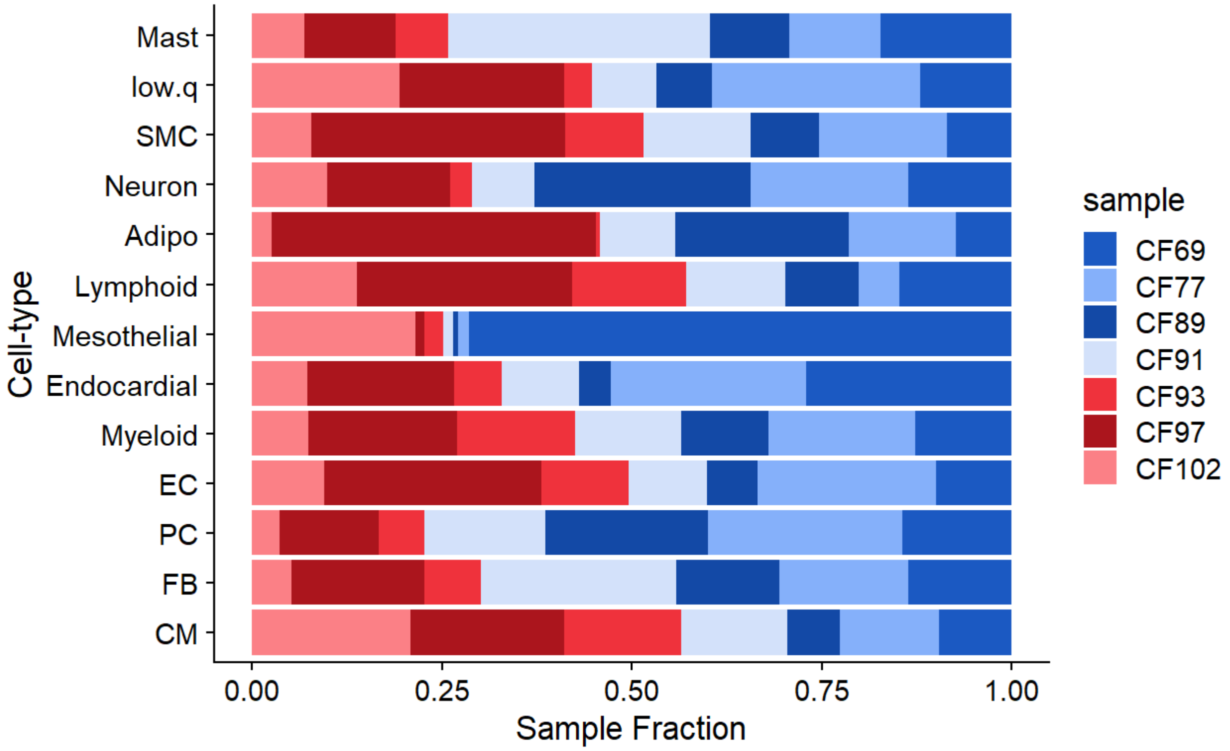

Supplementary Figure 5. Proportion of cell-types per donor. We see that all seven donors contributed cells of lower quality (low.q, high mitochondrial genes). We excluded these cells from downstream analyses. Adipo; Adipocytes, CM; Cardiomyocytes, EC; Endothelial cells, FB; Fibroblasts, PC; Pericytes, SMC; Smooth muscle cells.

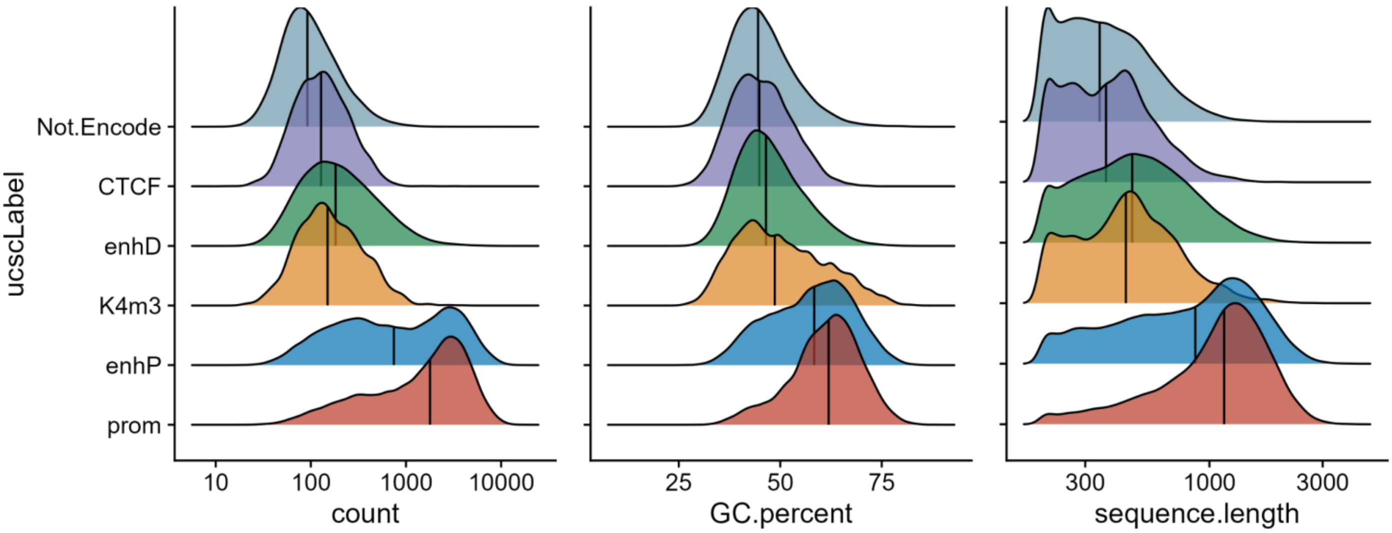

Supplementary Figure 6. Density plots of peak types based on their overlap with ENCODE candidate *cis*-regulatory elements (cCREs). From left to right, we show the distribution for each type of: (left) number of fragments per peak, (middle) the percentage of GC per peak, and (right) the length per peak. CTCF, CCCTC-binding factor mark; enhD, distal enhancer; enhP, proximal enhancer; K4m3, lysine 4 tri-methyl mark; not.Encode, peak found in the scAF dataset without any overlapping ENCODE peak; Prom, promoter.

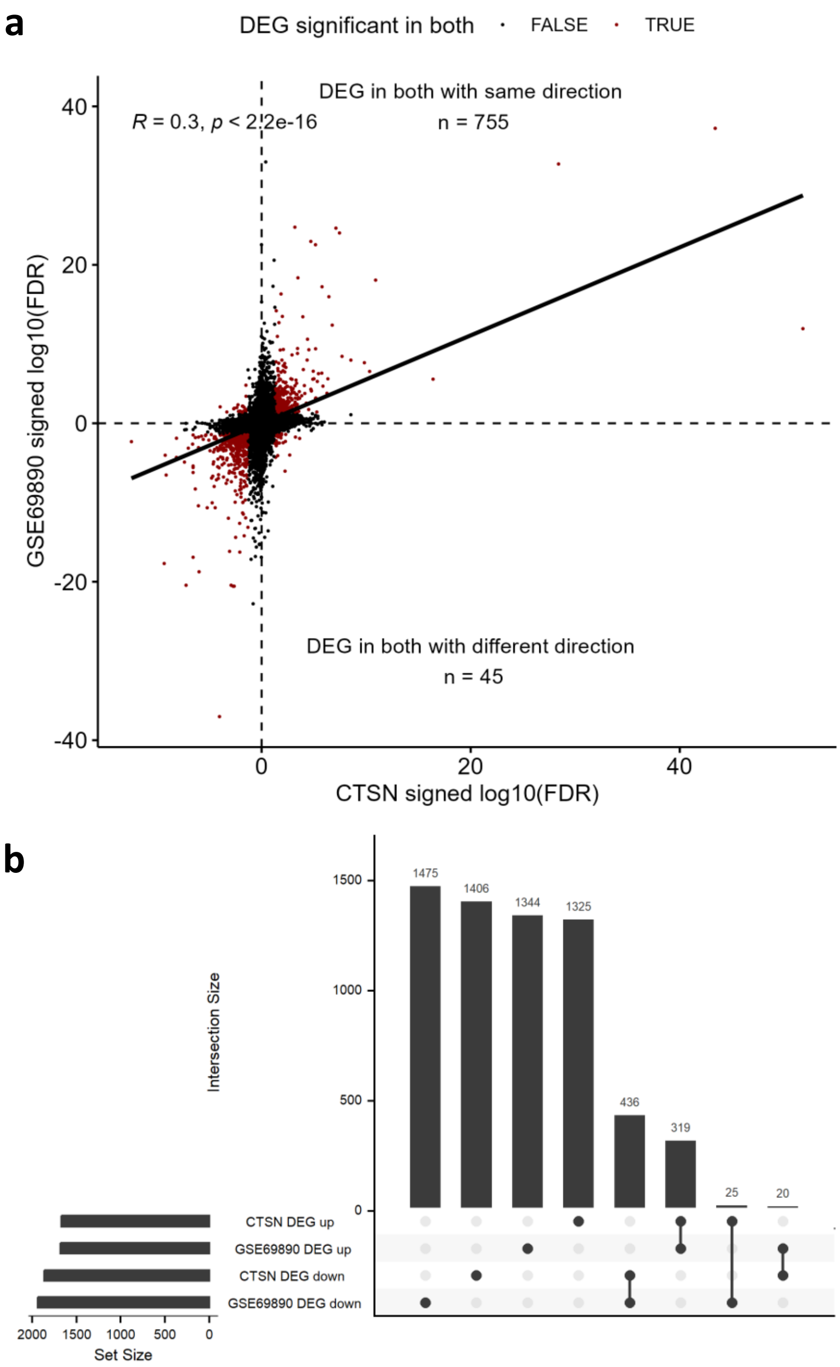

Supplementary Figure 7. Differential gene expression analyses between atrial fibrillation (AF) and sinus rhythm (SR) left atrial appendages (LAAs). (**a**) Scatter plot comparing the bulk RNAseq differential expression results in the CTSN and the GSE69890 datasets. The sign was attributed based on the $\log_2$ fold-change, with a positive sign indicating upregulation in AF. Red dots show differentially expressed genes (DEGs; false discovery rare [FDR] < 0.05 in both datasets). The Spearman's correlation rho and P-value are also shown. (**b**) Upset plot showing the number of genes in each set of intersecting DEGs. Up and down indicate upregulated and downregulated genes in AF.

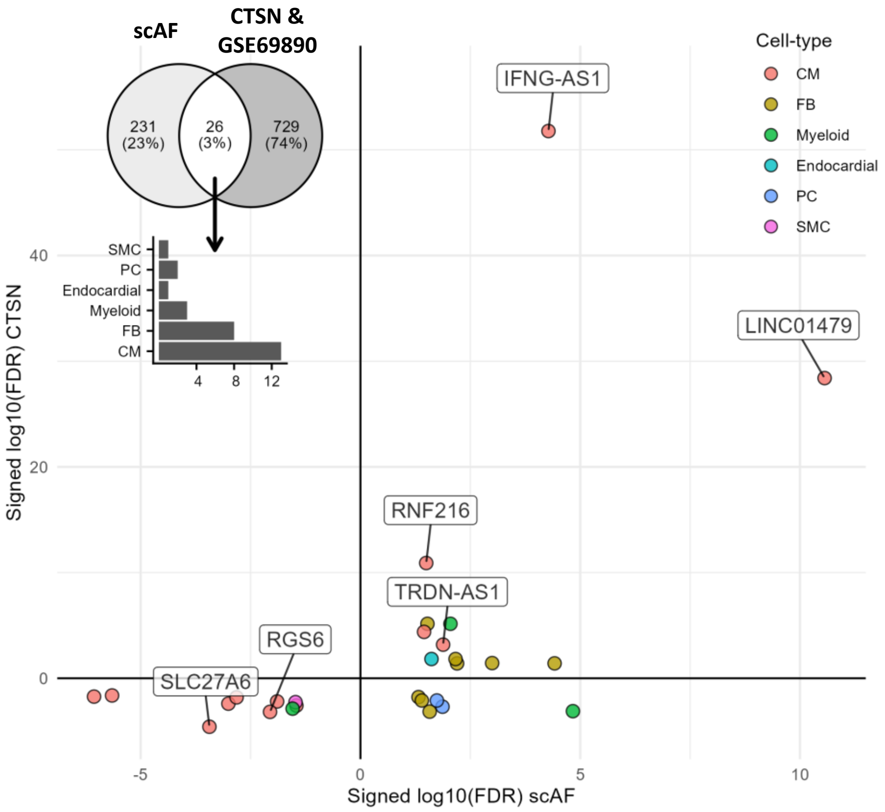

Supplementary Figure 8. Deconvolution of the left atrial appendage (LAA) bulk RNAseq data using the scAF dataset. Scatter plot comparing differential expression between atrial fibrillation (AF) and sinus rhythm (SR) samples in the CTSN and scAF datasets. Only genes that are differentially expressed in both bulk RNAseq datasets and in the scAF dataset are shown. The sign of the $\log_{10}$(false discovery rates [FDR]) is based on the direction of the $\log_2$ fold-change, with a positive value indicating upregulation in AF. Colors show the cell-type in which the gene was found to be differentially expressed in the scAF dataset. The inset Venn diagram shows the number of differentially expressed genes found in both the CTSN and GSE69890 datasets, and in the scAF dataset with the number of intersecting genes (26 genes). The bar plot below shows the number of differentially expressed genes found in this intersection by cell-types. CM; Cardiomyocytes, FB; Fibroblasts, PC; Pericytes, SMC; Smooth muscle cells.

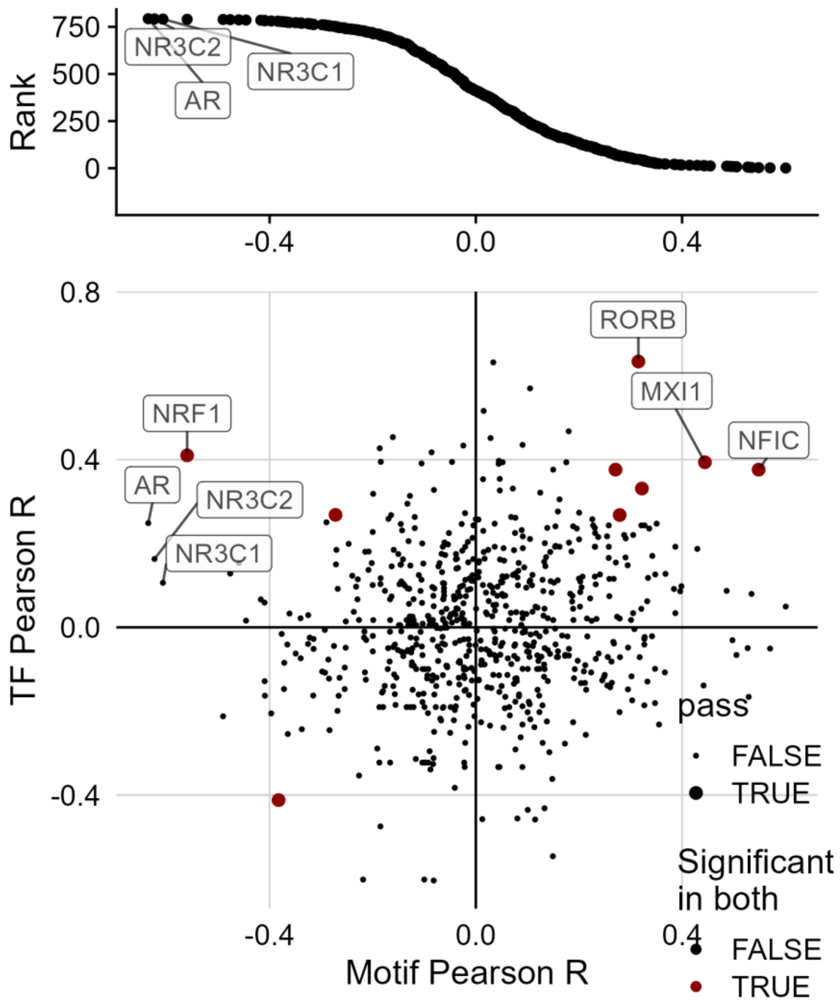

Supplementary Figure 9. Bulk RNAseq AF signatures correlation with transcription factor accessibility and expression in cardiomyocytes. (top) Rank plot showing the Pearson's correlation coefficient R for the motif activities correlation with the AF signature DOWN scores in cardiomyocyte (CM) meta-cells (**Methods**). (bottom) Scatter plot of the transcription factors (TF) and their motif activities correlation with the AF signature DOWN scores in CM meta-cells. Red dots represent TFs for which the expression and their motif activity is significantly correlated (false discovery rate <0.01) with the AF signature DOWN scores. AF, atrial fibrillation.

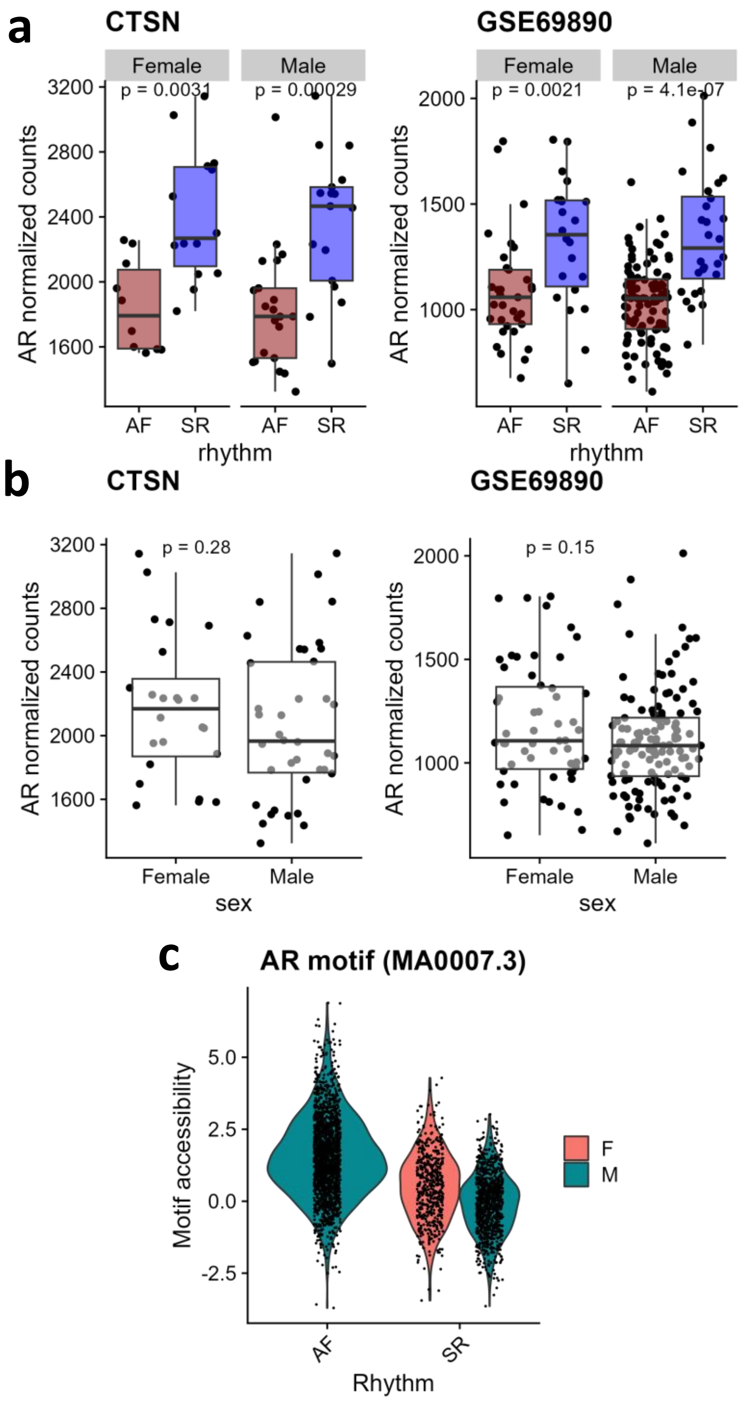

Supplementary Figure 10. AR expression and accessibility in males and females left atrial appendage samples from AF patients and SR controls. (**a-b**) Boxplots showing the normalized bulk RNAseq expression of AR in the CTSN and GSE69890 datasets. The box plots show the upper quartile, median and lower quartile. (**a**) Independent Wilcoxon test between AF and SR patients in males and females. (**b**) Wilcoxon test for AR expression between males and females. AR is systematically more expressed in SR controls, while being expressed at similar levels between male and female samples. (**c**) Violin plot showing the distribution of scAF AR motif accessibility of cardiomyocytes (JASPAR2020 motif MA0007.3) calculated with chromVAR in AF and SR patients, colored by patient sex (F; females [n=2], M; males [n=5]).

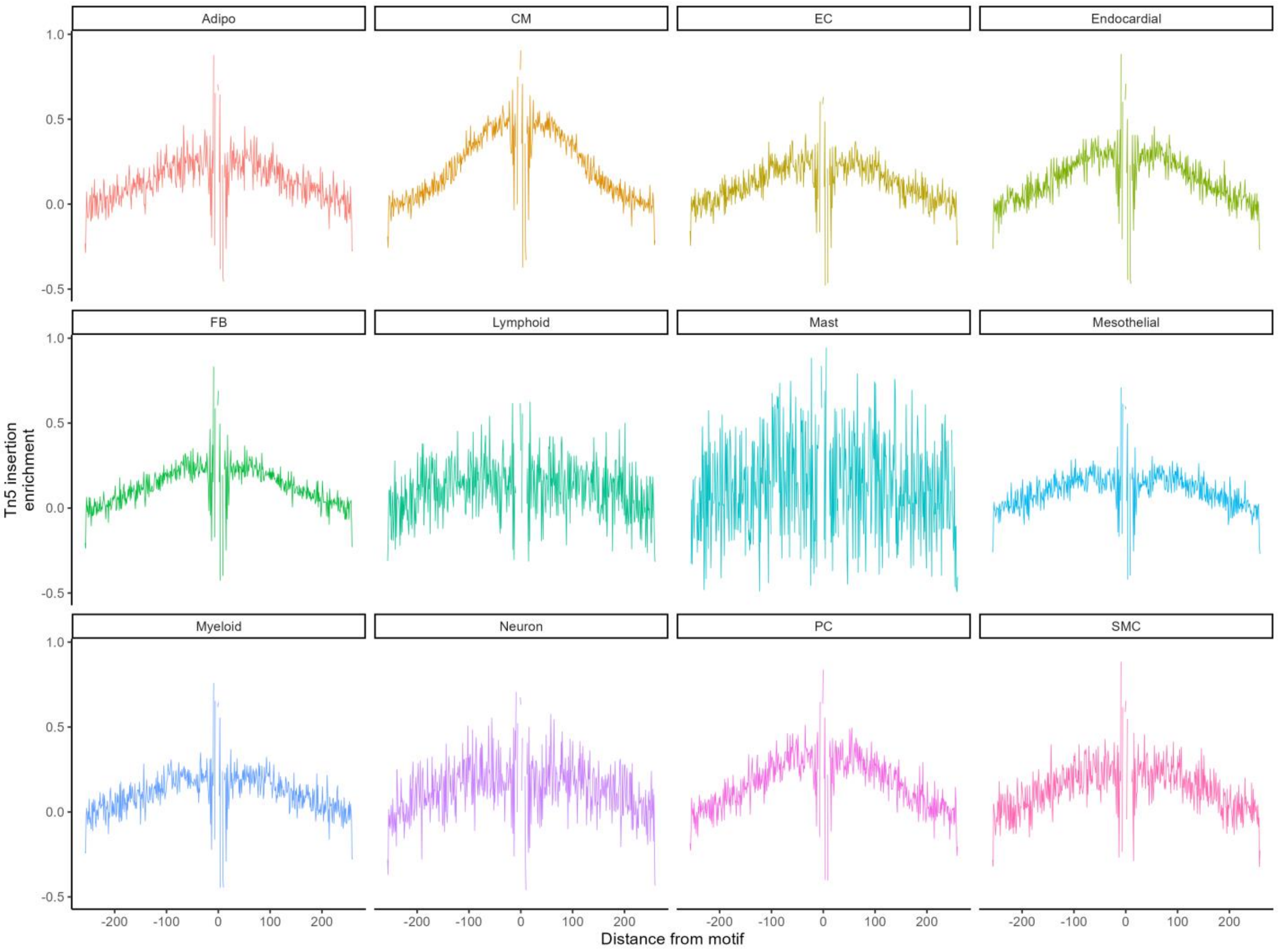

Supplementary Figure 11. Footprinting enrichments of the androgen receptor (AR) motif in each cell-type. Footprinting analysis of Tn5 insertions (ATACseq) within or near the AR binding motif of all left atrial appendage cell-types. CM; Cardiomyocytes, EC; Endothelial cells, FB; Fibroblasts, PC; Pericytes, SMC; smooth muscle cells.
