# Supplementary material for: Single-nucleus multi-omics implicates androgen receptor signaling in cardiomyocytes and NR4A1 regulation in fibroblasts during atrial fibrillation
Source: Nat Cardiovasc Res. 2025 Mar 25;4(4):433–44. doi: 10.1038/s44161-025-00626-0 (PMC11994452; doi:10.1038/s44161-025-00626-0)
Supplement: Supplementary file 2 — Reporting Summary [file 44161_2025_626_MOESM2_ESM.pdf]

nature portfolio

Corresponding author(s): Svetlana Reilly, Guillaume Lettre

Last updated by author(s): Feb 10, 2025

Reporting Summary

Nature Portfolio wishes to improve the reproducibility of the work that we publish. This form provides structure for consistency and transparency in reporting. For further information on Nature Portfolio policies, see our Editorial Policies and the Editorial Policy Checklist.

Statistics

For all statistical analyses, confirm that the following items are present in the figure legend, table legend, main text, or Methods section.

n/a	Confirmed	
☐	☒	The exact sample size (*n*) for each experimental group/condition, given as a discrete number and unit of measurement
☐	☒	A statement on whether measurements were taken from distinct samples or whether the same sample was measured repeatedly
☐	☒	The statistical test(s) used AND whether they are one- or two-sided<br>*Only common tests should be described solely by name; describe more complex techniques in the Methods section.*
☐	☒	A description of all covariates tested
☐	☒	A description of any assumptions or corrections, such as tests of normality and adjustment for multiple comparisons
☐	☒	A full description of the statistical parameters including central tendency (e.g. means) or other basic estimates (e.g. regression coefficient) AND variation (e.g. standard deviation) or associated estimates of uncertainty (e.g. confidence intervals)
☐	☒	For null hypothesis testing, the test statistic (e.g. *F*, *t*, *r*) with confidence intervals, effect sizes, degrees of freedom and *P* value noted<br>*Give P values as exact values whenever suitable.*
☒	☐	For Bayesian analysis, information on the choice of priors and Markov chain Monte Carlo settings
☐	☒	For hierarchical and complex designs, identification of the appropriate level for tests and full reporting of outcomes
☐	☒	Estimates of effect sizes (e.g. Cohen's *d*, Pearson's *r*), indicating how they were calculated

Our web collection on statistics for biologists contains articles on many of the points above.

Software and code

Policy information about availability of computer code

Data collection	No software used for data collection
Data analysis	R version 4.2.2 (packages: factoextra, presto, GenomicRanges); DESeq2 3.20 ; GraphPad Prism 10.1.2; Code at: https://github.com/lebf3/scAF_multiome

For manuscripts utilizing custom algorithms or software that are central to the research but not yet described in published literature, software must be made available to editors and reviewers. We strongly encourage code deposition in a community repository (e.g. GitHub). See the Nature Portfolio guidelines for submitting code & software for further information.

Data

Policy information about availability of data

All manuscripts must include a data availability statement. This statement should provide the following information, where applicable:

- Accession codes, unique identifiers, or web links for publicly available datasets
- A description of any restrictions on data availability
- For clinical datasets or third party data, please ensure that the statement adheres to our policy

The sn-multiome data discussed in this publication have been deposited in NCBI's Gene Expression Omnibus and are accessible through GEO Series accession number GSE238242 (https://www.ncbi.nlm.nih.gov/geo/query/acc.cgi?acc=GSE238242. The bulk RNAseq data for the CTSN cohort cannot be released on GEO but

is available from CTSN. Furthermore, we have released the CTSN bulk RNAseq gene count matrix and anonymized disease status information at: http://www.mhi-humangenetics.org/en/resources/. This information is sufficient to reproduce analyses described in this manuscript.

Research involving human participants, their data, or biological material

Policy information about studies with human participants or human data. See also policy information about sex, gender (identity/presentation), and sexual orientation and race, ethnicity and racism.

Reporting on sex and gender	Analyses were corrected for biological sex. Gender information was not available, and therefore we did not account for this in our analyses.
Reporting on race, ethnicity, or other socially relevant groupings	For the single-cell RNA-sequencing experiment, we did not consider race, ethnicity or genetic ancestry. For the CTSN bulk RNA-sequencing experiment, most participants were of European-ancestry.
Population characteristics	Population demographics are provided in Supplementary Tables 1 and 12 of the submitted manuscript.
Recruitment	Patients were recruited according to the approved study protocol, for example face-to-face communication with a trained researcher. All patients gave informed consent before enrolment and the signed consent form is stored securely. Patients scheduled for an elective cardiac surgery (valve repair/replacement or coronary artery bypass grafting, CABG, see Supplementary Tables 1 and 12) at the John Radcliffe Hospital were eligible for this study, unless they 1) underwent previous cardiac surgery in the last six months, 2) younger than 18 or older than 85 years of age, and 3) are not willing or not able to consent. There were no self-selection bias or other biases during patients recruitment.
Ethics oversight	Studies in human heart tissue and fibroblasts have been approved by the South Central-Berkshire B Research Ethics Committee (UK, REF: 18/SC/0404).

Note that full information on the approval of the study protocol must also be provided in the manuscript.

Field-specific reporting

Please select the one below that is the best fit for your research. If you are not sure, read the appropriate sections before making your selection.

☒ Life sciences ☐ Behavioural & social sciences ☐ Ecological, evolutionary & environmental sciences

For a reference copy of the document with all sections, see nature.com/documents/nr-reporting-summary-flat.pdf

Life sciences study design

All studies must disclose on these points even when the disclosure is negative.

Sample size	For the single-nucleus multiome experiment, we did not make power calculations to determine the right sample size. Indeed, power calculations for single-nucleus experiments are still unclear and depend on the expected effect sizes. In our single-cell experiment, we tried to maximize the number of donors, and the number of cells/donor. We also integrated our data with 4 publicly available single-nucleus RNAseq to increase power to identify cell-types, cell-type subgroups and differentially expressed transcriptomic modules.<br><br>For the bulk RNAseq data, we used 2 large publicly available datasets from human left atrial appendages (n=242 donors). We combined results and integrated them with the single-nucleus multiome data to minimize the risk to report false positive results.<br><br>Finally, throughout the article, we compare our results with the literature. When appropriate, we used published knowledge to validate our transcriptomic results, again indicating that our experiments are well-controlled for false positive results.
Data exclusions	There were no exclusions for most analyses. However, we noticed an outlier sample in our dataset, a SR donor who had a myocardial infarction, and hence, this sample was excluded from downstream analyses. This is mentioned and validated in the manuscript, can refer to Fig. 2c, Extended Data Fig. 6, and Extended Data Fig. 8. For the analysis on in vitro data, outliers were identified by ROUT test in GraphPad Prism and excluded.
Replication	For the single-nucleus RNAseq and ATACseq data from our experiments, we replicated the results using data from the Heart Atlas (single-cell RNAseq) and CATlas (single-nucleus ATACseq). We validated the cardiomyocyte and atrial fibrillation transcriptomic signatures using 4 publicly available single-nucleus RNAseq datasets.<br><br>For the bulk RNA-sequencing data, we used the intersection of both datasets to define the transcriptomic signatures. Therefore, results were validated internally (genes needed to be significant in both dataset, and assigned to a specific cell-type, to be in the signatures).<br><br>The in vitro findings were successfully replicated in at least two independent experiments. More details are provided:<br>Fig. 4. Data are pooled from individual donors assessed on the same day in one batch in single replicate (b,d,f,h,j,l,n,p) or duplicates (a,c,e,g,i,k,m) or triplicates (o). All results are reproduced independently for three times (a-b) in different subjects.<br>Extended Data Fig. 10. Data are pooled from individual donors assessed on the same day in one batch in single replicate (b,d,f,h,j,m,o,q,s,u) or duplicates (a,c,e,g,i,l,n,p,r,t) or triplicates (k,v).
Randomization	The data was not randomized as no treatments were tested.

Blinding	Blinding was applied to the researcher who generated the library. The atrial fibrillation status for the single-nucleus RNAseq data was only provided once once the libraries had been sequenced. Downstream analyses were not blinded as the researcher needs to know the grouping to perform bioinformatics analysis.

Reporting for specific materials, systems and methods

We require information from authors about some types of materials, experimental systems and methods used in many studies. Here, indicate whether each material, system or method listed is relevant to your study. If you are not sure if a list item applies to your research, read the appropriate section before selecting a response.

Materials & experimental systems

n/a	Involved in the study	
☐	☒	Antibodies
☒	☐	Eukaryotic cell lines
☒	☐	Palaeontology and archaeology
☒	☐	Animals and other organisms
☒	☐	Clinical data
☒	☐	Dual use research of concern
☒	☐	Plants

Methods

n/a	Involved in the study	
☒	☐	ChIP-seq
☒	☐	Flow cytometry
☒	☐	MRI-based neuroimaging

Antibodies

Antibodies used	GAPDH-HRP-conjugated G9295, Sigma, USA, 1/1000<br>NR4A1 (JM59-11) MA5-32647, Invitrogen, UK, 1/500<br>α-Smooth Muscle Actin A5228, Sigma, USA, 1/1000<br>Collagen I 234167, Sigma, USA, 1/2000<br>Collagen III alpha 1 NBP2-15946, Novus Biologicals, UK, 1/1000<br>Fibronectin F3648, Sigma, USA, 1/1000<br>Periostin 91771S, Cell Signaling Technology, USA, 1/1000
Validation	GAPDH-HRP-conjugated G9295 https://www.sigmaaldrich.com/catalog/product/sigma/g9295<br>NR4A1 (JM59-11) MA5-32647 https://www.thermofisher.com/antibody/product/NUR77-Antibody-clone-JM59-11-Recombinant-Monoclonal/MA5-32647<br>α-Smooth Muscle Actin A5228 https://www.sigmaaldrich.com/catalog/product/sigma/a5228<br>Collagen I 234167 https://www.sigmaaldrich.com/GB/en/product/mm/234167<br>Collagen III alpha 1 NBP2-15946 https://www.novusbio.com/products/collagen-iii-alpha-1-col3a1-antibody_nbp2-15946<br>Fibronectin F3648 https://www.sigmaaldrich.com/catalog/product/sigma/f3648<br>Periostin 91771S https://www.cellsignal.com/products/primary-antibodies/periostin-antibody/91771

Plants

Seed stocks	*Report on the source of all seed stocks or other plant material used. If applicable, state the seed stock centre and catalogue number. If plant specimens were collected from the field, describe the collection location, date and sampling procedures.*
Novel plant genotypes	*Describe the methods by which all novel plant genotypes were produced. This includes those generated by transgenic approaches, gene editing, chemical/radiation-based mutagenesis and hybridization. For transgenic lines, describe the transformation method, the number of independent lines analyzed and the generation upon which experiments were performed. For gene-edited lines, describe the editor used, the endogenous sequence targeted for editing, the targeting guide RNA sequence (if applicable) and how the editor was applied.*
Authentication	*Describe any authentication procedures for each seed stock used or novel genotype generated. Describe any experiments used to assess the effect of a mutation and, where applicable, how potential secondary effects (e.g. second site T-DNA insertions, mosiacism, off-target gene editing) were examined.*
