# Supplementary figures and images for: Single-nucleus multi-omics implicates androgen receptor signaling in cardiomyocytes and NR4A1 regulation in fibroblasts during atrial fibrillation
Source: Nat Cardiovasc Res. 2025 Mar 25;4(4):433–44. doi: 10.1038/s44161-025-00626-0 (PMC11994452; doi:10.1038/s44161-025-00626-0)

**Fig. 4b**

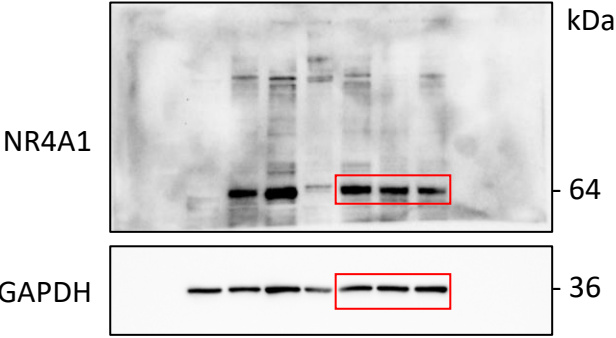

**Fig. 4d**

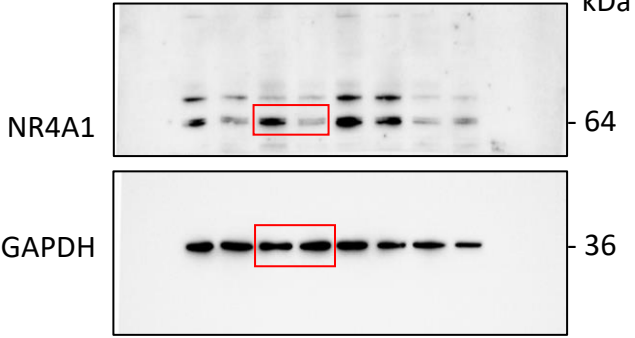

**Fig. 4f**

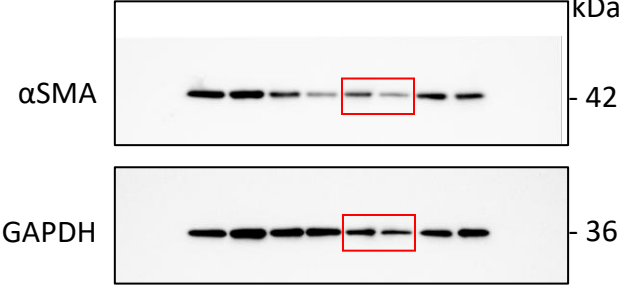

**Fig. 4h**

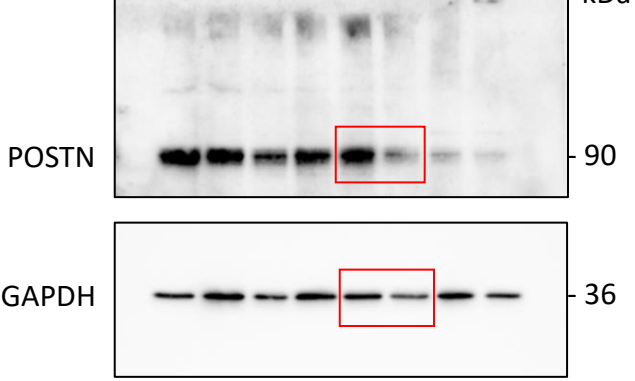

**Fig. 4j**

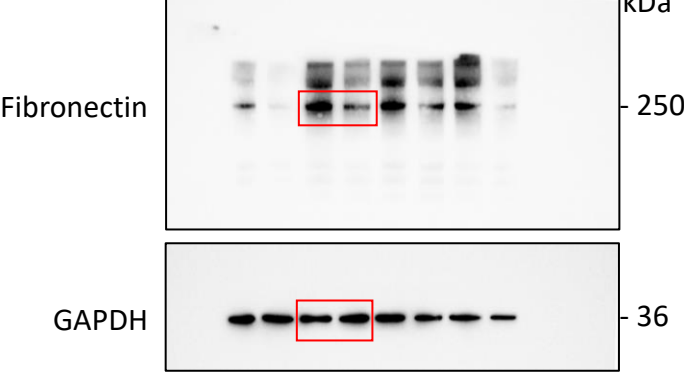

**Fig. 4l**

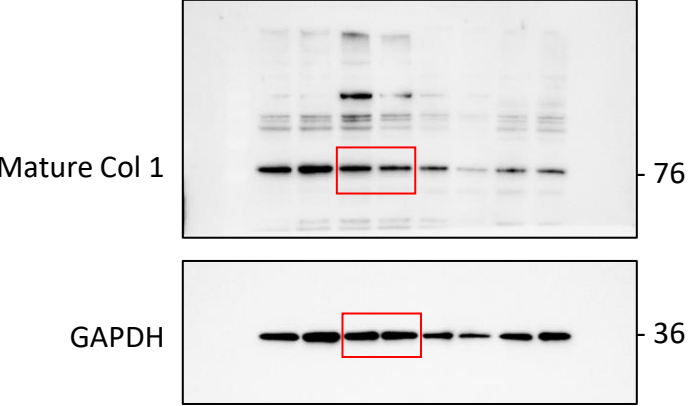

**Fig. 4n**

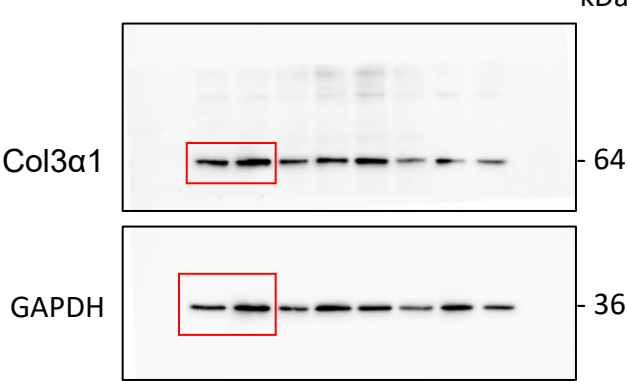

Supplement: Supplementary file 4 — Unprocessed western blots. [file 44161_2025_626_MOESM4_ESM.pdf]
