# Supplementary material for: Single-nucleus multi-omics implicates androgen receptor signaling in cardiomyocytes and NR4A1 regulation in fibroblasts during atrial fibrillation
Source: Nat Cardiovasc Res. 2025 Mar 25;4(4):433–44. doi: 10.1038/s44161-025-00626-0 (PMC11994452; doi:10.1038/s44161-025-00626-0)

Source Data – Extended Data Fig. 10b-10j

**Unmodified blots are identical as Source Data Fig.4 because this is a sub-analysis of the main dataset*

Extended Data Fig. 10b

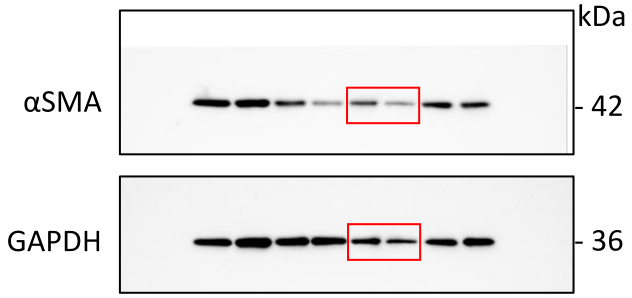

Extended Data Fig. 10d

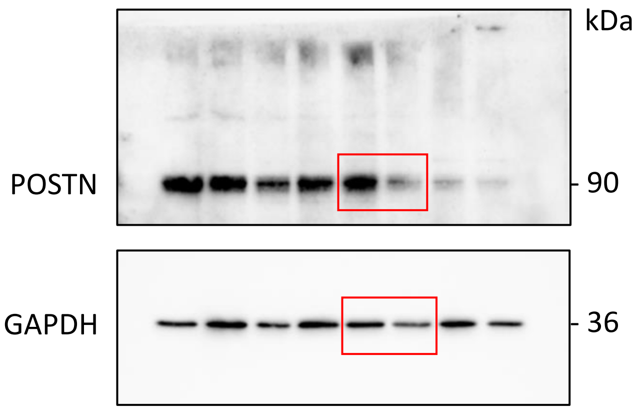

Extended Data Fig. 10f

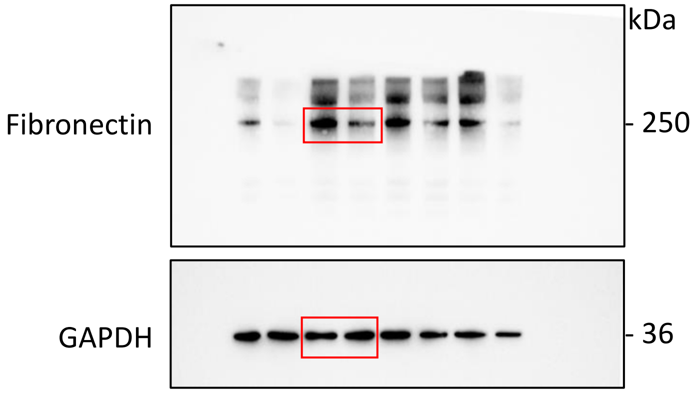

Extended Data Fig. 10h

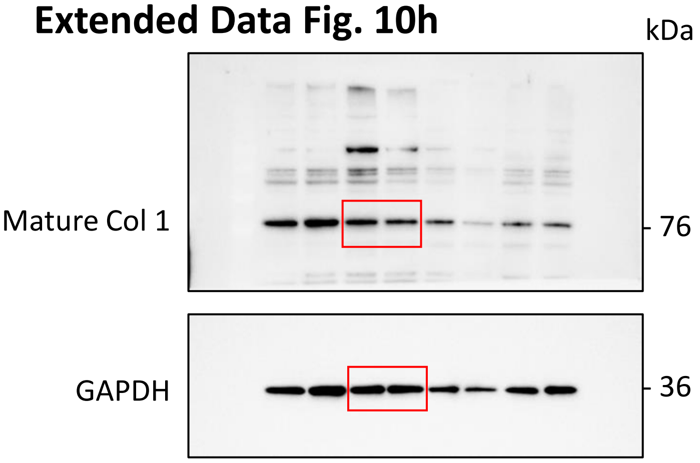

Extended Data Fig. 10j

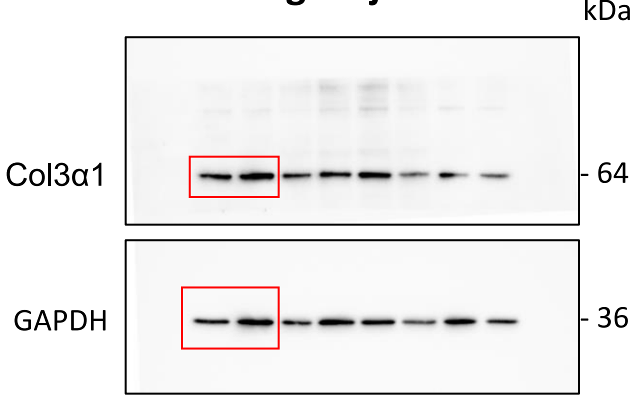

Source Data – Extended Data Fig. 10m-10u

Extended Data Fig. 10m

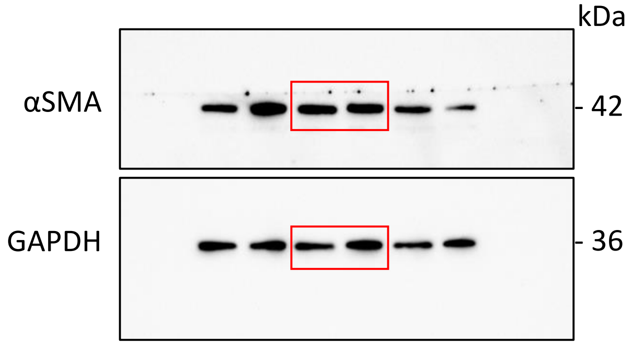

Extended Data Fig. 10o

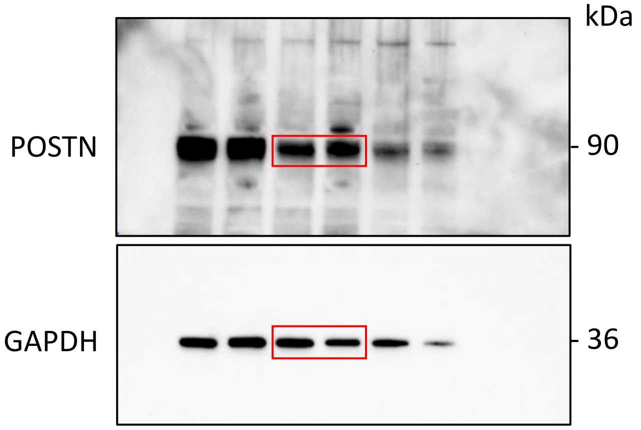

Extended Data Fig. 10q

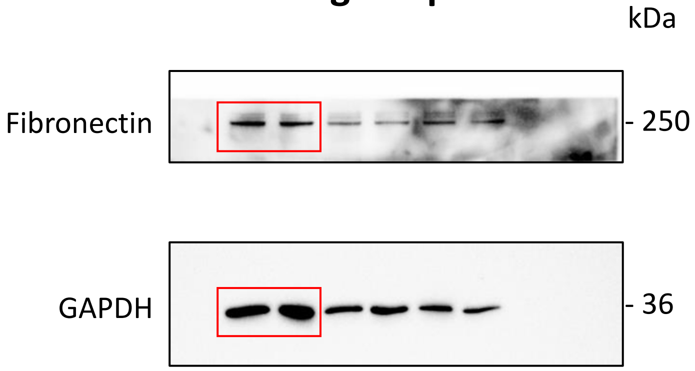

Extended Data Fig. 10s

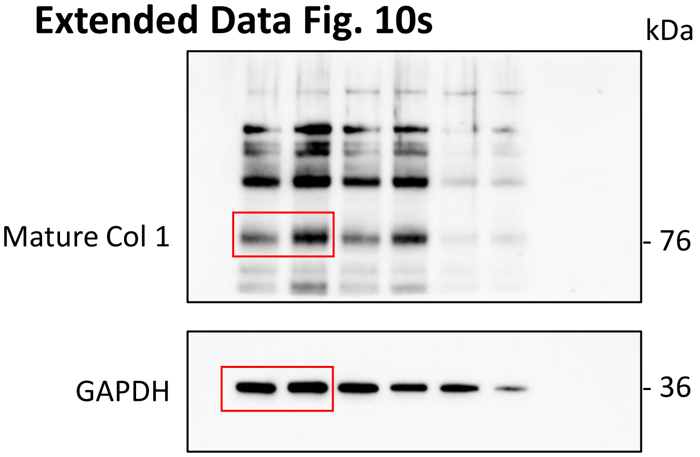

Extended Data Fig. 10u

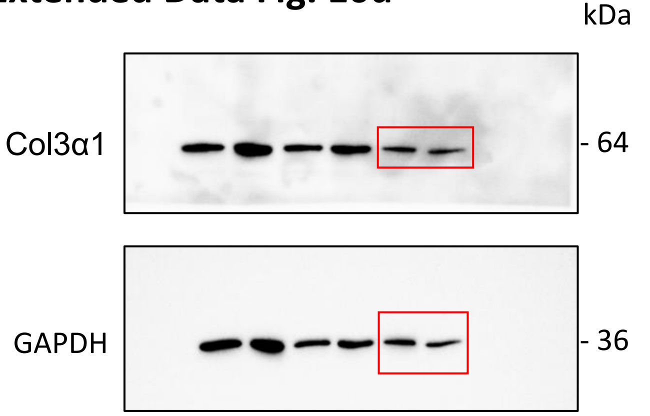

Supplement: Supplementary file 6 — Unprocessed western blots. [file 44161_2025_626_MOESM6_ESM.pdf]
